# A Conjugated Carboranyl Main Chain Polymer with Aggregation-Induced Emission in the Near-Infrared

**DOI:** 10.1021/jacs.4c03521

**Published:** 2024-05-06

**Authors:** Filip Aniés, Iain Hamilton, Catherine S. P. De Castro, Francesco Furlan, Adam V. Marsh, Weidong Xu, Valentina Pirela, Adil Patel, Michele Pompilio, Franco Cacialli, Jaime Martín, James R. Durrant, Frédéric Laquai, Nicola Gasparini, Donal D. C. Bradley, Martin Heeney

**Affiliations:** † Department of Chemistry, Centre for Processable Electronics, Molecular Sciences Research Hub, Imperial College London, 80 Wood Lane, London, W12 0BZ, U.K.; ‡ KAUST Solar Center, King Abdullah University of Science and Technology, Thuwal, 23955-6900, Saudi Arabia; § POLYMAT University of the Basque Country UPV/EHU, Av. de Tolosa 72, Donostia-San Sebastián, 20018, Spain; ∥ Department of Physics and Astronomy, London Centre for Nanotechnology, University College London, London, WC1E 6BT, U.K.; ⊥ Department of Engineering, Free University of Bozen-Bolzano, Università 5, Bolzano, I-39100, Italy; # Universidade da Coruña, Campus Industrial de Ferrol, CITENI, Esteiro, Ferrol, 15471, Spain; ○ NEOM Education, Research, and Innovation Foundation and University Neom, Al Khuraybah, Tabuk 49643-9136, Saudi Arabia

## Abstract

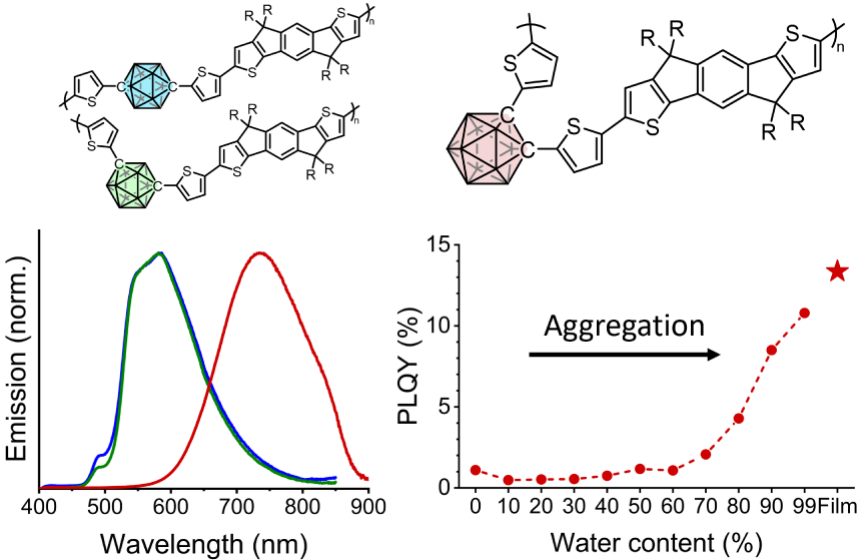

Materials exhibiting aggregation-induced emission (AIE) are both highly emissive in the solid state and prompt a strongly red-shifted emission and should therefore pose as good candidates toward emerging near-infrared (NIR) applications of organic semiconductors (OSCs). Despite this, very few AIE materials have been reported with significant emissivity past 700 nm. In this work, we elucidate the potential of *ortho*-carborane as an AIE-active component in the design of NIR-emitting OSCs. By incorporating *ortho*-carborane in the backbone of a conjugated polymer, a remarkable solid-state photoluminescence quantum yield of 13.4% is achieved, with a photoluminescence maximum of 734 nm. In contrast, the corresponding *para* and *meta* isomers exhibited aggregation-caused quenching. The materials are demonstrated for electronic applications through the fabrication of nondoped polymer light-emitting diodes. Devices employing the *ortho* isomer achieved nearly pure NIR emission, with 86% of emission at wavelengths longer than 700 nm and an electroluminescence maximum at 761 nm, producing a significant light output of 1.37 W sr^–1^ m^–2^.

## Introduction

Over the past few decades, the development of organic optoelectronic materials and devices, such as organic light-emitting diodes (OLEDs) and organic photovoltaics (OPVs), has benefitted from the fortunate overlap between visible light and the optical gap of most organic semiconductors (OSCs). As such, it is no surprise that focus has long been directed toward devices which operate within the visible range.^1^ That said, recent years have seen a surging interest in near-infrared (NIR) emitting materials and with good reason; with focus shifting toward new biomedical and *in vivo* applications, OSCs operating in the NIR region (700–2500 nm) are likely to play an important role.^2−5^ This is particularly true for the NIR-I region (700–950 nm), as this covers the first semitransparent window of biological tissue, enabling technologies such as biomedical imaging, biosensing, biometric technologies, and cancer therapy.^6−13^ Perovskite light emitting structures may also be of interest in this context.^14^

As for much of OSC research, material design is a key component in enabling NIR emissive devices.^15^ The development of such materials faces many challenges, the main one being the so-called “energy-gap law.”^16^ In essence, the energy-gap law states that a narrowing optical gap will increase the rate of nonradiative decay exponentially. As such, many fluorophores in the NIR region suffer from intrinsically low emissivity. Different approaches have been adopted to suppress or circumvent this issue. One early approach has been the use of phosphorescent organometallic complexes, where heavy metals provide access to stable lower-energy triplet states.^5,17^ The obvious drawback of such materials is their reliance on heavy metals, which often have toxicity issues that may complicate biomedical and *in vivo* applications.^4^ In recent years, other successful triplet-leveraging strategies have emerged, such as thermally activated delayed fluorescence (TADF).^18^ TADF relies on reverse intersystem crossing and can be achieved using purely organic molecules. However, few TADF materials succeed by combining high photoluminescence quantum yield (PLQY) with photoluminescence maxima (λ_PL_) greater than 700 nm.^19,20^

Importantly, neither of the aforementioned strategies addresses the second challenge in designing NIR emitters: aggregation-caused quenching (ACQ). Upon aggregation of organic fluorophores, a dramatic loss in PLQY is typically seen, resulting from nonradiative decay pathways enabled through intermolecular π–π interactions. This is particularly true for low-optical gap materials, which typically require extended and highly rigid conjugated systems, thus facilitating π–π overlap. As a countermeasure, OLED fabrication often relies on host matrices to suppress aggregation and facilitate charge transfer. This entails other drawbacks, however, such as increasing device complexity and instability, and typically results in blue-shifting of the emission.^21 −25^

One approach which could potentially address this is aggregation-induced emission (AIE).^26,27^ Contrary to ACQ, excited AIE-active species undergo nonradiative relaxation in solution and attain fluorescent properties upon aggregation through restriction of intramolecular motion.^28,29^ This requires a molecular design, which prevents both intramolecular motion and intermolecular π–π interactions in the solid state – e.g. through a high degree of rotational disorder.^30,31^ Although the red-shifted nature of the AIE emission makes such materials excellent candidates for NIR emission, AIE materials with significant emission past 700 nm are few and far between.^32−40^ One reason may be that the most common AIE components – such as tetraphenylethene – rely on highly twisted structures, preventing extended conjugation and narrow optical gap structures.^31^

Over the past 15 years, icosahedral *ortho*-carborane has surfaced as an AIE-active moiety when coupled to various conjugated structures.^41−43^ In contrast to twisted moieties, its AIE mechanism originates from nonradiative vibrations in the carborane carbon–carbon (C_C_–C_C_) bond, which are suppressed upon aggregation. Meanwhile, overlap between substituted π-conjugated systems and the C_C_–C_C_ σ* orbital enables the formation of radiative low-energy intramolecular charge transfer (ICT) excited states, inducing a red-shifted emission in the aggregated state.^44−47^ Consequentially, AIE properties are unique to carbon substituted *ortho*-carborane, whereas *meta* and *para* isomers or boron-substituted species remain susceptible to ACQ.^48,49^ Critically, carboranyl compounds have also been proven safe for medicinal treatments such as boron neutron capture therapy, demonstrating their potential for *in vivo* applications.^50^

Despite the widespread interest in carboranyl emitters, only a couple of NIR emissive compounds have been reported, and they suffer from very low PLQY.^51,52^ Likewise, there are surprisingly few accounts of carboranyl compounds in OLED devices.^53−59^ In this work, we utilize the AIE properties of a polymer (*o*CbT_2_-IDT) incorporating *ortho*-carborane directly in the conjugated backbone to achieve a λ_PL_ at 734 nm and a remarkable solid-state PLQY of 13.4%. Furthermore, we observe contrasting behavior in the respective *para* and *meta* isomers (*p*CbT_2_-IDT and *m*CbT_2_-IDT), which emit in the visible region and undergo ACQ. Finally, we demonstrate the applicability of all three polymers as the emissive material in nondoped OLEDs. Most notably, highly radiant OLEDs based on the *ortho* isomer are shown to emit nearly pure NIR emission with an electroluminescence maximum (λ_EL_) at 761 nm and 86% of the emission greater than 700 nm. This establishes *ortho*-carborane as an important building block when designing new NIR emissive AIE polymers for optoelectronic applications.

## Results and Discussion

### Material Design and Characterization

**Scheme 1:**
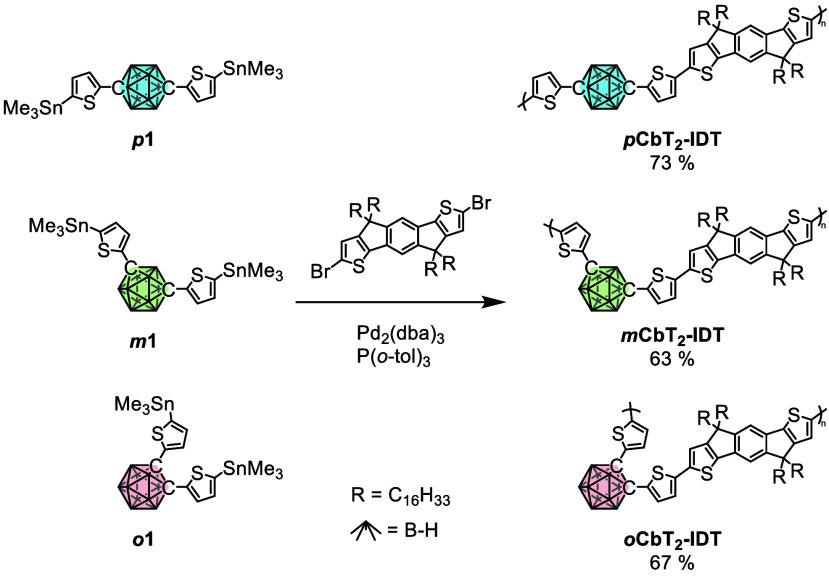
Synthesis of Target Polymers

The synthesis of the three polymers presented in this work is depicted in Scheme 1. In pursuit of a narrow optical gap material, indacenodithiophene (IDT) was chosen as the central aromatic moiety due to its extended π-conjugated core and high density solubilizing side chain.^60,61^ Furthermore, its frequent use as an electron donating moiety makes it well suited to promote efficient ICT with the carboranyl unit.^62,63^ A polymeric structure was chosen for the benefit of processability and quality of fabricated devices.^64^ This was achieved by coupling the dibrominated IDT core with di(5-(trimethylstannyl)thiophen-2-yl)-carborane comonomers under Stille polymerization conditions using microwave heating.^65,66^ Here, the flanking thiophenes play a dual role; while facilitating the polymerization, they also extend the conjugated π-system, further narrowing the optical gap of the final polymer. Following polymerization, the polymers were isolated by precipitation followed by sequential solvent extraction with methanol, acetone, and hexane. All polymers were extracted into hexane, with good solubility related to the long (hexadecyl) side chains on the IDT comonomer.

Polymer weights were estimated using analytical gel permeation chromatography (GPC) against a polystyrene standard in 80 °C chlorobenzene, as outlined in Table 1. From this data, it is clear that the choice of carborane isomer has an impact on the degree of polymerization, as the number average polymer weight (*M*_n_) of the *p*CbT_2_-IDT polymer was approximately twice as large as that of *m*CbT_2_-IDT, which in turn was approximately twice that of *o*CbT_2_-IDT. Although the accuracy of the estimated weights may be affected by the different polymeric shapes, there is a clear trend, which may be explained in terms of steric hindrance. As the substituted carborane carbons are positioned closer to each other, there will be more steric hindrance around the reactive center in the polymerization step, possibly inhibiting reaction rates and final polymer lengths. This hypothesis is supported by comparison with our recent work, where copolymerization with the bulkier naphthalene diimide (NDI) core resulted in a further reduction of molecular weights, but with a similar isomeric trend.^65^

**Table 1:** Polymer Weights, as Measured by GPC against Polystyrene Standards, and Thermal Data of Each Polymer

polymer	*M*_n_ (kDa)	*M*_w_ (kDa)	*Đ*	*T*_d,5%_ (°C)	*R*_w,700 °C_ (%)
*p*CbT_2_-IDT	73	191	2.59	410	39
*m*CbT_2_-IDT	36	89	2.47	406	36
*o*CbT_2_-IDT	19	35	1.92	407	41

To investigate the thermal properties of the polymers, thermal gravimetric analysis (TGA) and differential scanning calorimetry (DSC) were performed, with results summarized in Table 1. TGA running at 5 °C/min under nitrogen revealed near identical degradation behavior between the polymers, with degradation temperatures *T*_d,5%_ marking a 5% weight loss at 406–410 °C (Figure S1a). Likewise, DSC scans showed no difference between polymers, with no signs of thermal transitions between 25 and 350 °C when scanning at 10 °C/min, suggesting an amorphous polymeric structure (Figure S1b).

To further investigate the microstructure of the polymers, atomic force microscopy (AFM) was used to image surface topography of spin-coated thin films, as seen in Figure S2. All films appeared very smooth, with as-cast *p*CbT_2_-IDT slightly rougher than *m*CbT_2_-IDT and *o*CbT_2_-IDT, with root-mean-square (RMS) values of 0.203, 0.150, and 0.166 nm, respectively. The amorphous morphology was further confirmed by grazing-incidence wide-angle X-ray scattering (GIWAXS) diffractograms of polymer films (Figure S3). Only one broad isotropic pattern was observed, which is associated with diffuse scattering from nonperiodic molecular packing, confirming a disordered morphology. Neither AFM nor GIWAXS detected any significant effects of annealing the films at 100 °C for 10 min. While the kinked polymeric structure of *m*CbT_2_-IDT and *o*CbT_2_-IDT can help to explain the disordered morphology, this should not apply to the linear *p*CbT_2_-IDT. However, the high degree of rotational freedom between the carboranyl unit and appended thiophenes may disrupt the planarity of the polymer backbone. Accordingly, density functional theory (DFT) calculations revealed low rotational energetic barriers at 0.3 kcal/mol and a negligible difference (<0.03 kcal/mol) between the five stable conformations (Figure S4). Consequentially, the polymer chain may adopt a variety of rotational conformations with no particular preference for either of the five stable states and thus lead to a disordered morphology.

### Electronic Properties

**Figure 1:**
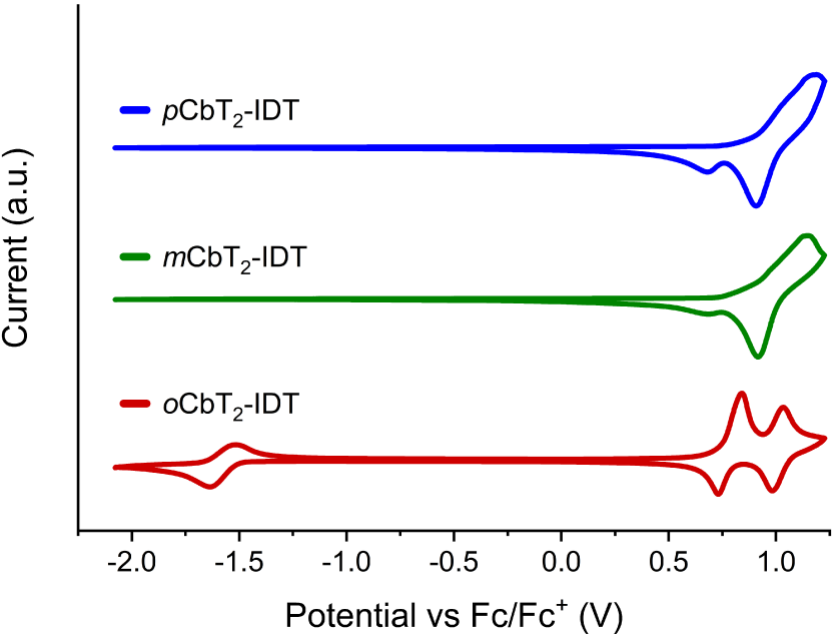
Cyclic voltammograms of drop-cast films of each polymer.

The electrochemical properties of the polymers were investigated using cyclic voltammetry (CV). Polymers were drop-cast onto a working electrode, and scans were referenced against the ferrocene/ferrocenium (Fc/Fc^+^) couple. Scans of *p*CbT_2_-IDT and *m*CbT_2_-IDT (Figure 1) appeared nearly identical, with oxidation potentials derived from the peak onsets at 0.77 and 0.75 V respectively. Meanwhile, *o*CbT_2_-IDT behaved differently, with two distinct reversible oxidation peaks at 0.74 and 0.92 V. A possible explanation is that the polar *ortho*-carborane stabilizes the oxidized aromatic moiety, resulting in two very distinct electrochemical events. Meanwhile, *para* and *meta* carboranes offer no such stabilization, resulting in more diffuse peaks, as is often seen in conjugated polymers. It also gave rise to one reversible reduction peak, with an onset of −1.51 V, which can be attributed to the less negative reduction potential of *ortho*-carborane compared to the *meta* and *para* isomers.^67^ Assuming 4.8 eV for the Fc/Fc^+^ couple, this results in ionization potential values of 5.57, 5.55, and 5.54 eV for *p*CbT_2_-IDT, *m*CbT_2_-IDT, and *o*CbT_2_-IDT, respectively, and an electron affinity of 3.29 eV for *o*CbT_2_-IDT.

Theoretical energy values of the frontier molecular orbitals (FMOs) were calculated using DFT on the B3LYP/6-311G(d,p) level of theory. For computational feasibility, trimers were modeled for each species, with methyl replacing alkyl chains. In all three polymers, the highest occupied molecular orbitals (HOMOs) and lowest unoccupied molecular orbitals (LUMOs) were located on the conjugated IDT and thiophene moieties, with carboranes acting as “conjugation breakers” (Figure S5). This feature was also reported for a similar NDI-based n-type polymer.^65^ As is summarized in Table 2, calculated FMO energy levels were similar between *p*CbT_2_-IDT and *m*CbT_2_-IDT, with a slightly deeper HOMO of *m*CbT_2_-IDT. Meanwhile, the HOMO of *o*CbT_2_-IDT was 0.16 eV lower than that of *m*CbT_2_-IDT, and the LUMO 0.38 eV lower, reflecting the more electron withdrawing nature of the *ortho*-carborane. The calculated FMOs were all found at shallower levels than those measured experimentally. Furthermore, the calculated energy gaps range between 2.70 and 2.92 eV, which are wider than those derived from the absorption onset at 2.21–2.25 eV (Figure 3a). Such discrepancies are well documented in the literature and have been explained by several factors such as solvation and polarization effects, and the fundamental differences between bulk properties and modeling of individual molecules in vacuum.^68,69^

**Table 2:** Energetic Estimates as Obtained through Computational and Experimental Methods

	HOMO (eV)		LUMO (eV)		optical gap (eV)	
polymer	DFT	CV^a^	DFT	CV^a^	DFT	UV–vis
*p*CbT_2_-IDT	–5.11	–5.57	–2.25	-	2.86	2.25
*m*CbT_2_-IDT	–5.18	–5.55	–2.26	-	2.92	2.25
*o*CbT_2_-IDT	–5.34	–5.54	–2.64	–3.29	2.70	2.21

a Estimated from the redox peak onset.

### Photophysical Properties

**Figure 2:**
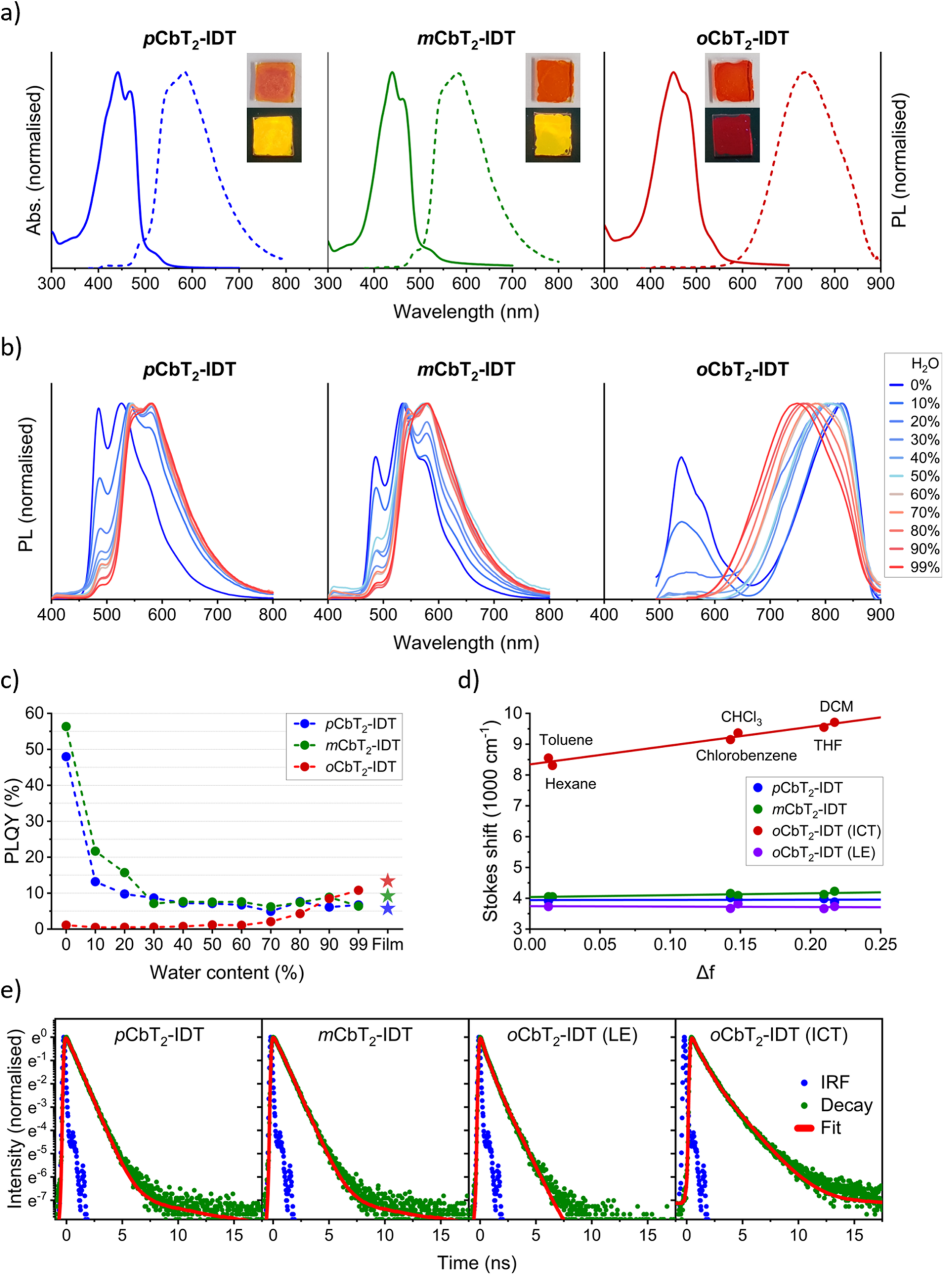
(a) Absorption (solid line) and PL (dashed line) spectra of films of respective polymer. Inset: photos of films under ambient (top) and UV (bottom) light. λ_ex_ = 375 nm. (b) PL spectra of THF solutions (≤0.005 mg/mL) with varying fractions of water. (c) PLQY of THF:water solutions (≤0.005 mg/mL) and thin films of respective polymer. (d) Lippert–Mataga plot derived from absorption and PL spectra of polymers in different solvents. LE and ICT peaks of *o*CbT_2_-IDT are plotted separately. (e) TCSPC plots (logarithmic intensity scale) and fits of polymers dissolved in THF, measured at 540 nm. The ICT state of *o*CbT_2_-IDT was measured from a 1:9 solution of THF:water at 750 nm. λ_ex_ = 467 nm. IRF = instrument response function.

As an initial assessment of the emissive properties of the polymers, absorption and emission spectra were measured for spin-coated thin films, as depicted in Figure 2a. Absorption spectra were similar between polymers, with absorption maxima (λ_abs_) at 442 and 439 nm for *p*CbT_2_-IDT and *m*CbT_2_-IDT, respectively, and a slight redshift of *o*CbT_2_-IDT at 450 nm. The emission spectra of *p*CbT_2_-IDT and *m*CbT_2_-IDT were nearly identical, with λ_em_ = 582 and 581 nm, respectively. On the other hand, *o*CbT_2_-IDT exhibited a large red-shift, resulting in NIR emission with a peak maximum at λ_em_ = 734 nm. The resulting Stokes shift (between absorption and emission peaks) was large at 8598 cm^–1^, compared to 5442 and 5567 cm^–1^ for *p*CbT_2_-IDT and *m*CbT_2_-IDT, respectively.

To further investigate the different behaviors between the polymers, emission spectra were recorded for polymer solutions. Starting with pure THF, continuous fractions of water (0–99 vol %) as antisolvent were used as a way of controlling polymer aggregation.^30^ As seen in Figure 2b, this prompted major changes in the *o*CbT_2_-IDT spectrum. With increasing water fractions, the high-energy peak at λ_em_ = 539 nm gradually faded, coupled with an enhancement of the low-energy peak and a blue-shift from λ_em_ = 830 to 749 nm, strongly indicating AIE properties. Meanwhile, *p*CbT_2_-IDT and *m*CbT_2_-IDT spectra did not shift significantly, although the intensity of the lower-energy shoulder increased. This is likely an effect of polymer aggregation resulting in scattering and self-absorbance. No changes were seen in the absorption spectra for either polymer, except for a slight decrease in shoulder peak intensities (Figure S6).

To quantify the emission, PLQYs of solutions and thin films were measured, as plotted in Figure 2c and summarized in Table 3.^70^ THF solutions of *p*CbT_2_-IDT and *m*CbT_2_-IDT were strongly emissive with PLQY values of 48.0 and 56.4%, respectively. Upon addition of water, however, the PLQYs dropped to a plateau between 5 and 10%. This could be observed visually (Figure S7) and is clear evidence of ACQ behavior in these polymers.

**Table 3:** Photophysical Data of Polymers

polymer	λ_em_^a^ (nm)	φ_THF_^b^ (%)	φ_film_^b^ (%)	<τ> (ns)
*p*CbT_2_-IDT	582^c^	48.0	5.7	0.87^d^
*m*CbT_2_-IDT	581^c^	56.4	9.2	0.92^d^
*o*CbT_2_-IDT (LE)	539^d^	1.1	-	0.61^d^
*o*CbT_2_-IDT (ICT)	734^c^	-	13.4	1.0^e^

a λ_ex_ = 375 nm.

b λ_ex_ = 361 nm.

c Measured from films.

d Measured from THF solution.

e Measured from 1:9 THF:water solution.

Meanwhile, *o*CbT_2_-IDT dissolved in THF was only very weakly emissive, with PLQYs measured at ≲1% up to a water concentration of 60%. Upon solvation in a 3:7 THF:water mixture, however, the PLQY was doubled to 2.1%, followed by a gradual increase up to 10.8% PLQY at 99% water content, thus confirming AIE behavior. Moreover, the solid-state PLQY was measured at 13.4%, which is remarkable considering the low energy of the emission. Notably, this is higher than both *p*CbT_2_-IDT and *m*CbT_2_-IDT in the solid state, measured at 5.7% and 9.2%, respectively.

To trace the origin of the emissive behaviors, solvatochromic properties were investigated in six different solvents: toluene, hexane, chlorobenzene, chloroform, THF, and DCM (Figure S8). In the case of *o*CbT_2_-IDT, the emission spectra changed markedly between solvents; the low-energy peak experienced a red-shift with increasing solvent polarity, while the high-energy peak was static. Meanwhile, almost no differences could be discerned in solutions of *p*CbT_2_-IDT and *m*CbT_2_-IDT. Two exceptions can be found in the PL spectra of *p*CbT_2_-IDT in hexane and DCM, most probably as a consequence of poor solubility, which was observed in these solutions. This may be traced to the *para* isomer, since a carboranyl isomer with lesser exposure to the environment seems to decrease solubility.^65^ The reader is reminded, however, that the choice of carboranyl isomer also affects polymer weight, which may in turn impact solubility as well. The overall lower solubility of *p*CbT_2_-IDT may explain its lower emissivity in THF, compared to *m*CbT_2_-IDT.

To illustrate the different solvatochromic behaviors, a Lippert–Mataga plot was constructed plotting Stokes shifts against solvent polarity, as seen in Figure 2d. Solvent polarity Δ*f* was defined using the equation
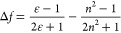
1where ε is the dielectric constant, and *n* is the refractive index (parameters used for each solvent are listed in Table S1).^71^ Since a polar excited species will undergo solvent relaxation in a polar environment – leading to lower-energy emission – the slope of the fitted line is related to the transition dipole moment, i.e., the difference between the excited state and the ground state. As can be seen, fitted lines of *p*CbT_2_-IDT, *m*CbT_2_-IDT, and the high-energy *o*CbT_2_-IDT peak are almost flat, indicating a very small transition dipole moment, as would be the case for a locally excited (LE) state. Meanwhile, the low-energy *o*CbT_2_-IDT peak has a much steeper slope, thus confirming a larger transition dipole moment, as is expected for a polar ICT excited state.^72^

Time-correlated single-photon counting (TCSPC) was used to measure fluorescence lifetimes of the LE states of polymers dissolved in THF, as well as the ICT state of *o*CbT_2_-IDT when dissolved in a 1:9 THF:water solution. The resulting lifetimes are summarized in Tables 3 and S2, and fitted plots are shown in Figure 2e. The short lifetimes between 0.61 and 1.0 ns indicate fluorescent emission in all solutions.^73,74^*p*CbT_2_-IDT and *m*CbT_2_-IDT are dominated by one decay component, while the *o*CbT_2_-IDT decay is seemingly biexponential. The average lifetime of the *o*CbT_2_-IDT LE state was considerably shorter than those of *p*CbT_2_-IDT and *m*CbT_2_-IDT. This is likely a consequence of faster nonradiative decay due to vibrations of the C_C_–C_C_ bond.^46,75^ Indeed, considering the correlation
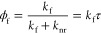
2the rate constant for the nonradiative decay *k*_nr_ can be determined as 6.7 × 10^8^, 4.7 × 10^8^, and 1.6 × 10^9^ s^–1^ for *p*CbT_2_-IDT, *m*CbT_2_-IDT, and *o*CbT_2_-IDT LE states, respectively. Meanwhile, the lifetime of the *o*CbT_2_-IDT ICT state in 1:9 THF:water was longer and the derived *k*_nr_ lower (9.2 × 10^8^ s^–1^). This provides evidence for the predicted AIE mechanism of the *ortho*-carboranyl polymer, where aggregation results in suppression of fast C_C_–C_C_ vibrational decay and thereby promotes ICT and the subsequent red-shifted radiative decay.^44,47,76^

### Organic Light-Emitting Diodes

Nondoped polymer OLEDs were fabricated in order to evaluate the applicability of carborane-containing polymers in NIR and light emitting devices. OLEDs were fabricated by solution processing using the device structure ITO/PEDOT:PSS/TFB/CbT_2_-IDT/TPBi/LiF/Al, where each of the three polymers was used as the emissive layer. Here, poly(3,4-ethylenedioxythiophene) polystyrene sulfonate (PEDOT:PSS) is used as the hole transporting layer on the indium tin oxide (ITO) anode, and poly(9,9-dioctylfluorene-*alt*-*N*-(4-sec-butylphenyl)-diphenylamine) (TFB) is used as an electron blocking layer to reduce electron leakage, as well as reducing exciton quenching between the hole transporting layer and the emissive layer.^77^ 2,2′,2″-(1,3,5-benzinetriyl)-tris(1-phenyl-1-*H*-benzimidazole) (TPBi) is used as both an electron transporting layer and hole blocking layer, and a thin LiF electron injection layer was deposited before the aluminum cathode. Device performance parameters are summarized in Table 4, and low turn-on voltages – below 4 V in all devices – indicate an appropriate choice of charge transport layers resulting in low charge injection barriers.

**Table 4:** Device Data of Fabricated OLEDs, and Hole Mobilities Extracted from SCLC Devices

	λ_EL_ (nm)	*V*_on_ (V)	Lum._max_ (cd m^–2^)	Rad._max_ (mW sr^–1^ m^–2^)	luminance eff. (cd/A)	power eff. (lm/W)	EQE (%)	μ_h_ (cm V^–1^ s^–1^)
*p*CbT_2_-IDT	544	3.7	1555	-	0.52 (4.0 V)	0.40 (4.0 V)	0.18 (4.4 V)	1.5 × 10^–7^
*m*CbT_2_-IDT	543	3.5	2763	-	0.94 (4.0 V)	0.75 (3.8 V)	0.29 (4.2 V)	2.1 × 10^–5^
*o*CbT_2_-IDT	761	3.8	-	1366	-	-	0.10 (4.2 V)	2.4 × 10^–5^

**Figure 3:**
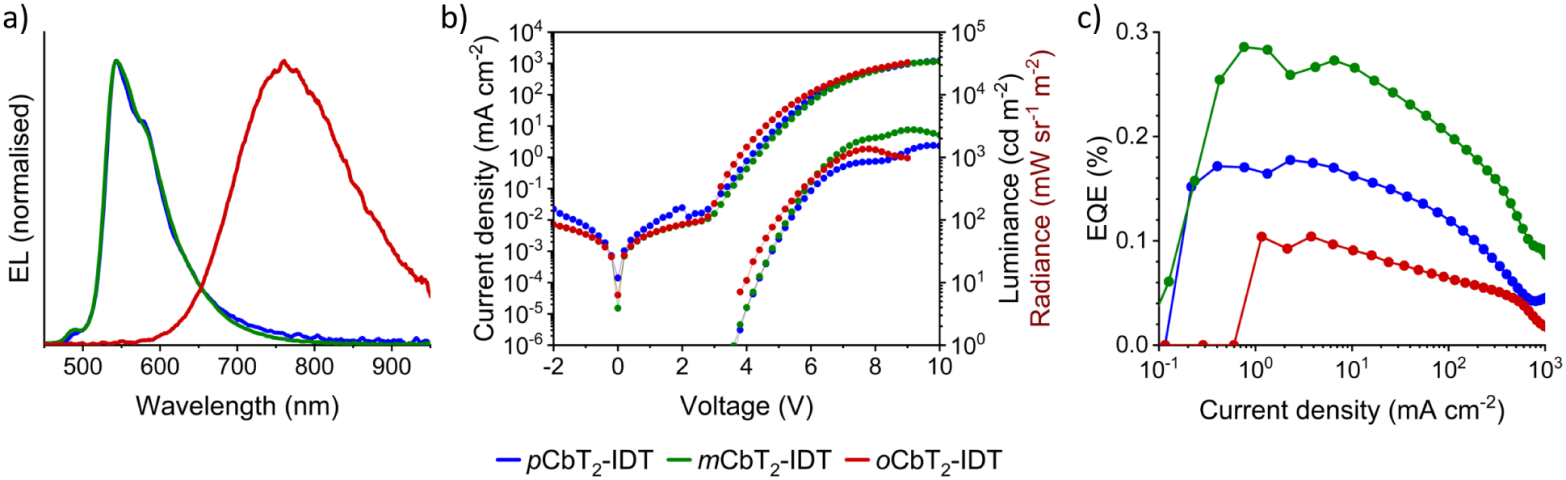
(a) Electroluminescence spectra of fabricated OLEDs. The spectra for *p*CbT_2_-IDT and *m*CbT_2_-IDT closely overlap. (b) *J*–*V*–*L* plots for *p*CbT_2_-IDT and *m*CbT_2_-IDT and *J*–*V*–*R* plot for *o*CbT_2_-IDT (note different right axis units). (c) OLED EQE values plotted against current density.

Figure 3a depicts the electroluminescence (EL) spectra of the devices. Devices employing *p*CbT_2_-IDT and *m*CbT_2_-IDT exhibit nearly identical spectra, with electroluminescence maxima (λ_EL_) of 544 and 543 nm, respectively. These visible light OLEDs were very bright, with maximum luminance values of 1555 and 2763 cd m^–2^ for *p*CbT_2_-IDT and *m*CbT_2_-IDT, respectively (Figure 3b). It is likely that these devices benefit from the nondoped structure in that the emission is neither shifted nor inhibited by a host matrix. Meanwhile, *o*CbT_2_-IDT exhibits almost pure NIR emission with a λ_EL_ = 761 nm peak, EL extending well-beyond 950 nm, and 86% of emission at wavelengths longer than 700 nm. Furthermore, a high radiance of 1.37 W sr^–1^ m^–2^ was obtained from these NIR OLEDs.

The fact that these devices are based on fluorescent materials prompts a maximum external quantum efficiency (EQE) of ∼5% due to spin selection rules and outcoupling limitations.^1^ Although the efficiency of the NIR emitting OLED is in line with many previously reported fluorescent NIR devices, the overall low EQE points toward nonidealities in the charge balance of the emissive layers.^2^ To investigate this further, hole carrier mobilities were extracted from fabricated space-charge-limited current (SCLC) devices (Figure S9, Table 4). The lower hole mobility of *p*CbT_2_-IDT compared to *m*CbT_2_-IDT, with 1.5 × 10^–7^ and 2.1 × 10^–5^ cm^2^ V^–1^ s^–1^, respectively, may explain its lower EQE despite otherwise very similar material and device characteristics, as this may affect the charge transfer balance in the device. Meanwhile, the lower EQE of *o*CbT_2_-IDT is far more likely to originate from the difference in physical properties for this material, not the least considering the very similar mobility to *m*CbT^2^-IDT at 2.4 × 10^–5^ cm^2^ V^–1^ s^–1^. One possible cause is the heavily red-shifted emission of the material. Considering that the typical OLED has a weak microcavity structure with a reflecting aluminum back electrode, device layer thicknesses should ideally be optimized so that the emitted light escapes the device where there is an antinode.^78^ Hence longer wavelength emission calls for thicker device layers. Therefore, the device structure would need to be significantly altered to achieve improved optical outcoupling, which is a key loss driver in OLED efficiency.^79^

## Conclusion

In conclusion, three novel fluorescent carboranyl polymers have been synthesized – *p*CbT_2_-IDT, *m*CbT_2_-IDT, and *o*CbT_2_-IDT – incorporating each of the three icosahedral carboranyl isomers in the polymer backbone. The *ortho*-carboranyl polymer exhibited clear AIE properties, resulting in a remarkable 13.4% solid-state PLQY and a λ_PL_ of 734 nm. This behavior is traced to restricted C_C_–C_C_ vibrations upon aggregation, reducing nonradiative decay and thus enhancing a radiative ICT excited state, as is evident through solvatochromic observations and excitation lifetime measurements. In contrast, *para* and *meta* isomers were strongly emissive in solution but exhibited suppressive ACQ upon aggregation. The potential of all three polymers for electronic applications was demonstrated through the fabrication of nondoped OLEDs, where the polymers were used in a single-component emissive layer. Leveraging the AIE properties of *o*CbT_2_-IDT, a highly radiant device emitting near pure NIR light was achieved, with 86% of emission above 700 nm and an λ_EL_ peak at 761 nm, covering the entire NIR-I region. Devices based on *p*CbT_2_-IDT and *m*CbT_2_-IDT exhibited similar characteristics to each other, with brilliant emission in the 500–700 nm range. Overall, these devices demonstrate the potential of carboranyl materials for light-emitting applications in general and for NIR emitting OLEDs in particular.

## Supporting Information Available

Supporting figures and tables, experimental details, and chemical analytical data; TGA and DSC scans of the polymer (Figure S1); AFM images of the pristine and annealed polymer films (Figure S2); 2D GIWAXS and cross-sectional diffractograms (Figure S3); DFT calculated energetic barrier of rotation around the carborane-thiophene bond of a single *p*CbT_2_-IDT repeat unit (Figure S4); DFT modelling of the molecular structures and frontier molecular orbitals (Figure S5); absorption spectra of THF:water solutions (Figure S6); photographs of vials containing THF:water solutions (Figure S7); absorption and PL spectra of polymer solutions (Figure S8); output plots for SCLC devices (Figure S9); ^1^H NMR, ^11^B NMR, FTIR spectra, and analytical GPC trace and peak report of *p*CbT_2_-IDT (Figures S9–S13), *m*CbT_2_-IDT (Figures S14–S17), and *o*CbT_2_-IDT (Figures S18–S21) (PDF)
