# Supplementary material for: A Conjugated Carboranyl Main Chain Polymer with Aggregation-Induced Emission in the Near-Infrared
Source: J Am Chem Soc. 2024 May 6;146(19):13607–16. doi: 10.1021/jacs.4c03521 (PMC11100012; doi:10.1021/jacs.4c03521)
Supplement: Supplementary file 1 — ja4c03521_si_001.pdf [file ja4c03521_si_001.pdf]

Supporting Information

A conjugated carboranyl main chain polymer with aggregation-induced emission in the near-infrared

Filip Aniés,[a] Iain Hamilton,[b] Catherine S. P. De Castro,[b] Francesco Furlan,[a] Adam V. Marsh,[b] Weidong Xu,[a] Valentina Pirela,[c] Adil Patel,[d] Michele Pompilio,[d] Franco Cacialli,[d,e] Jaime Martín,[f] James R. Durrant,[a] Frédéric Laquai,[b] Nicola Gasparini,[a] Donal D. C. Bradley,[b,g] Martin Heeney*[a,b]

[a] Department of Chemistry
Centre for Processable Electronics
Molecular Sciences Research Hub
Imperial College London
London W12 0BZ
U.K.

[b] KAUST Solar Center
King Abdullah University of Science and Technology
Thuwal 23955-6900
Saudi Arabia

[c] POLYMAT
University of the Basque Country UPV/EHU
Donostia-San Sebastián 20018
Spain

[d] Department of Physics and Astronomy
London Centre for Nanotechnology
University College London
London WC1E 6BT
U.K.

[e] Department of Engineering
Free University of Bozen-Bolzano
Bolzano I-39100
Italy

[f] Universidade da Coruña
Campus Industrial 507 de Ferrol
CITENI
Esteiro
Ferrol 15471
Spain

[g] NEOM Education, Research, and Innovation Foundation and University Neom
Al Khuraybah
Tabuk 49643-9136
Saudi Arabia

Characterization procedures

Atomic force microscopy (AFM) images of spin coated films (10 mg/mL solutions in chlorobenzene, 1000 rpm for 1 min) were obtained with an Agilent AFM 5500 setup in tapping mode, and processed with PicoView 1.5 software.

Thermogravimetric analysis (TGA) was run on a Mettler Toledo TGA/DSC 1 from 25 to 750 °C with a heating rate of 5 °C/min under nitrogen.

Differential scanning calorimetry (DSC) measurements were performed on a Mettler Toledo DSC 1 over three scans between 25 and 350 °C with a heating rate of 10 °C/min.

Cyclic voltammetry (CV) was performed using an Autolab PGSTAT101 potentiostat with a glassy carbon working electrode, Pt counter electrode, and Ag/Ag^+ reference electrode in a 0.1 M solution of tetrabutylammonium hexafluorophosphate in acetonitrile, at a scan rate of 0.1 V/s. Samples were drop-cast onto the working electrode before conducting each measurement and ferrocene was added at the end of the measurements as an internal reference.

Analytical gel permeation chromatography (GPC) was performed at 80 °C on an Agilent Technologies 1200 series chromatograph equipped with a refractive index detector, running in chlorobenzene with two PLgel mixed B columns in series. The system was calibrated against narrow polydispersity polystyrene standards.

Computational models of each polymer, using trimers with methyl groups in place of alkyl chains for the sake of computational feasibility, were optimised with Gaussian 16 (revision C.01) at the B3LYP/6-311G** level.[1,2] The same level of theory was used for rotational energy calculations. Molecular structures and orbitals were visualised in GaussView 5.0.

Grazing-incidence wide-angle X-ray scattering (GIWAXS) measurements were performed at the BL11 NCD-SWEET at ALBA Synchrotron Radiation Facility (Barcelona, Spain). The incident X-ray beam energy was set to 12.4 eV using a channel cut Si (1 1 1) monochromator. The angle of incidence α_i was set between 0.1° and 0.2° to ensure surface sensitivity. Data are expressed as a function of the scattering vector, which was calibrated using Cr_2O_3, obtaining a sample-to-detector distance of 145.6 mm. The scattering patterns were recorded using a Rayonix LX255-HS area detector, which consists of a pixel array of 1920 × 5760 pixels (H × V) with a pixel size of 44 × 44 μm^2. All the measurements were performed under N_2 atmosphere to minimize the damage of the films. 2D GIWAXS patterns were corrected as a function of the components of the scattering vector (q).

The steady-state photoluminescence (PL) emission spectra were measured with a Jobin Yvon Fluorolog spectrofluorometer from Horiba. The samples were excited using Xe lamp source and detected using PMT-Si detector with an extended correction range up to 1000 nm. The films were excited at 375 nm and appropriate combinations of bandpass and longpass filters were used to avoid 2nd order diffraction. The emission spectra were corrected for the spectral sensitivity of the setup corresponding to the used configuration (detectors and gratings). The PL quantum yield (PLQY) of the solutions was recorded through the relative method with either Coumarin 6 or Nile Blue A in ethanol (3.6 μM and 1.2 μM, respectively) as the standard (Φ_F= 0.78 and 0.27, respectively).[3–6] All solutions were carefully optically matched by using a Cary 5000 UV-Vis-NIR spectrophotometer and measured under the same conditions. The refractive indexes of solvent mixtures were obtained from literature and respective polynomial fitting equation for the 99% mixture.[7] For solid-state PLQY measurements, films were spin coated onto clean glass substrates (cleaning procedure outlined under device section below) from toluene solutions (5 mg/mL) at 2000 rpm for 60 seconds, followed by annealing at 100 °C for 10 min. PLQY was measured in air and obtained by the absolute method using a Labsphere integrating sphere with the same Horiba fluorescence spectrometer and compared with both Nile Blue A and Cresyl Violate Perchlorate 650 in ethanol (Φ_F= 0.27 and 0.56, respectively).[8–10] The monochromatic beam was incident at an angle of 60° to the film and emission was collected at the same angle. Solvatochromic absorption and PL spectra were measured on a Cary 60 UV-Vis Spectrophotometer, and a Cary Eclipse Fluorescence Spectrometer (λ_{ex} = 375 nm), respectively.

Device fabrication

OLEDs

Patterned indium tin oxide (ITO)-on-glass substrates (size 25.4 mm × 25.4 mm) were cleaned in a succession of ultrasonic baths using acetone, isopropanol, and detergent (Hellmanex III, 2 vol% DI water) for 15 min each, followed by UV ozone treatment in an MTI UV/Ozone ProCleaner. A 35 nm layer of PEDOT:PSS (AI 4083 from Heraeus) was deposited by spin coating at 3000 rpm and then annealed in nitrogen for 15 min at 135 °C followed by a 12 nm layer of TFB spin-coated from a 2 mg/mL toluene solution at 1000 rpm for 30 s. The TFB layer was annealed at 180 °C for 60 min in nitrogen. A 35 nm emissive layer was deposited via spin coating from a 5 mg/mL solution in toluene at 2000 rpm. Finally, the devices were transferred to an Angstrom glovebox evaporator where 30 nm of TPBi, 1 nm of LiF and 100 nm of Al were sequentially evaporated at a pressure of 1×10^{-7} mbar. The devices were encapsulated and characterised in air using a Hamamatsu external quantum efficiency measurement system C9920-12 in conjunction with a Keithley 2400 sourcemeter and controlled using a PC.

SCLC

Hole-only devices were fabricated onto patterned ITO-coated glass. Prior to deposition, substrates were cleaned by sonication in detergent solution, water, acetone, and isopropyl alcohol for 15 min each. Devices were fabricated with the configuration ITO/PEDOT:PSS/CbT_2-IDT/MoOx(10nm)/Ag. PEDOT:PSS was filtered and spin-coated onto the plasma-treated substrates to achieve ~50 nm thickness and annealed at 150°C for 15 min, followed by deposition of 110 nm active layers from chlorobenzene solutions at 2000 rpm under inert atmosphere. Layers of 10 nm MoO_x and 100 nm Ag were subsequently deposited by evaporation through a shadow mask with pixel areas of 0.045 cm^2. Active layer thicknesses were measured with a Dektak profilometer.

Figures and tables

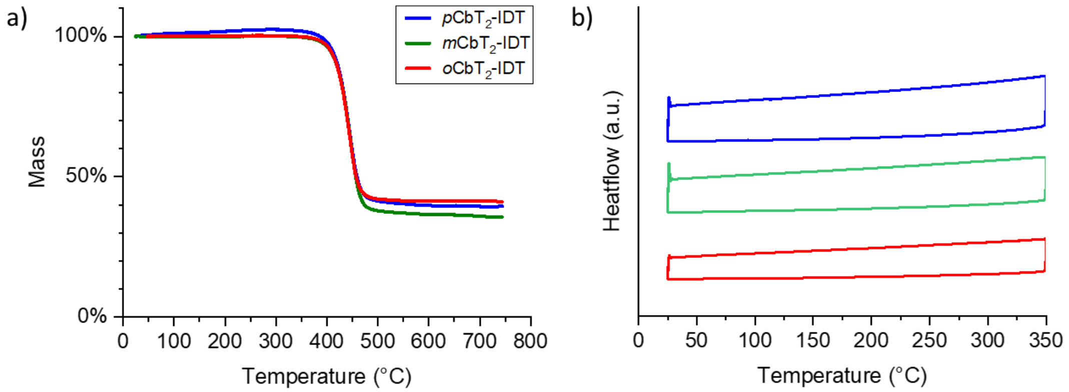

Figure S1. a) TGA and b) DSC scans of polymers.

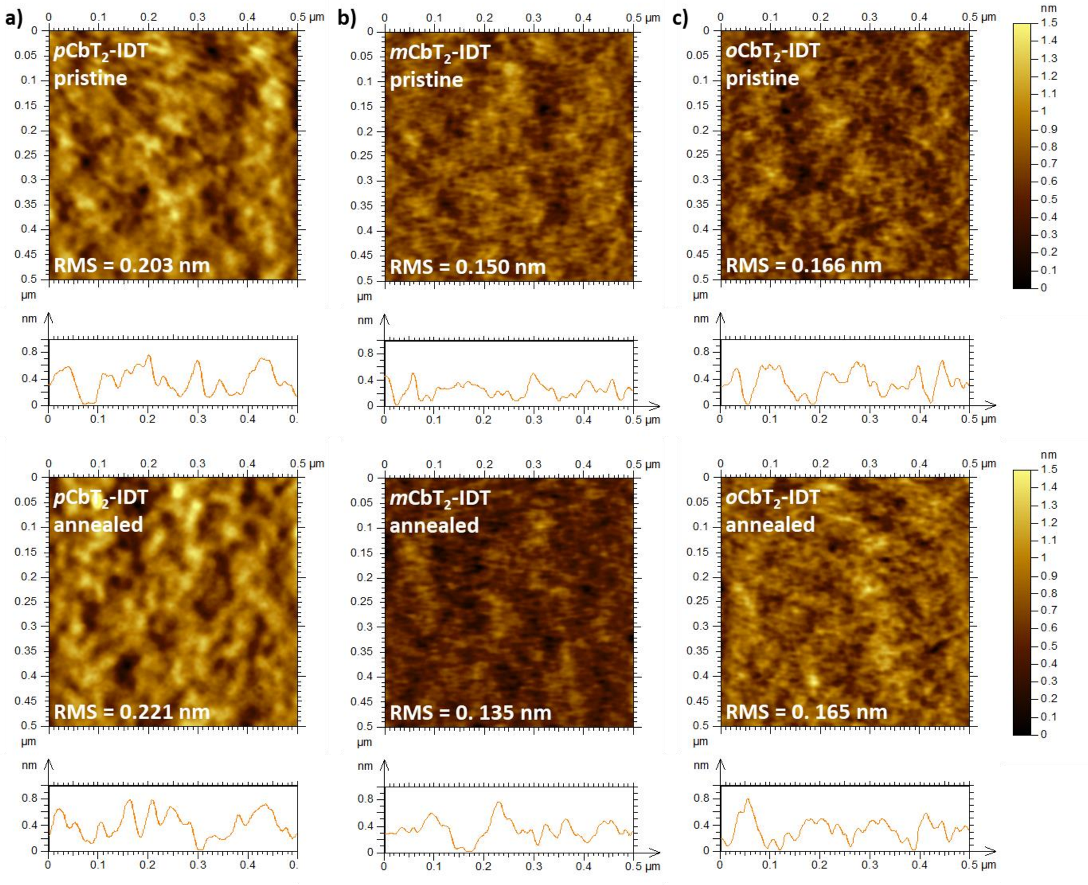

Figure S2. AFM images of the pristine (top) and annealed (bottom) polymer films of a) *p*CbT2-IDT, b) *m*CbT2-IDT, and c) *o*CbT2-IDT.

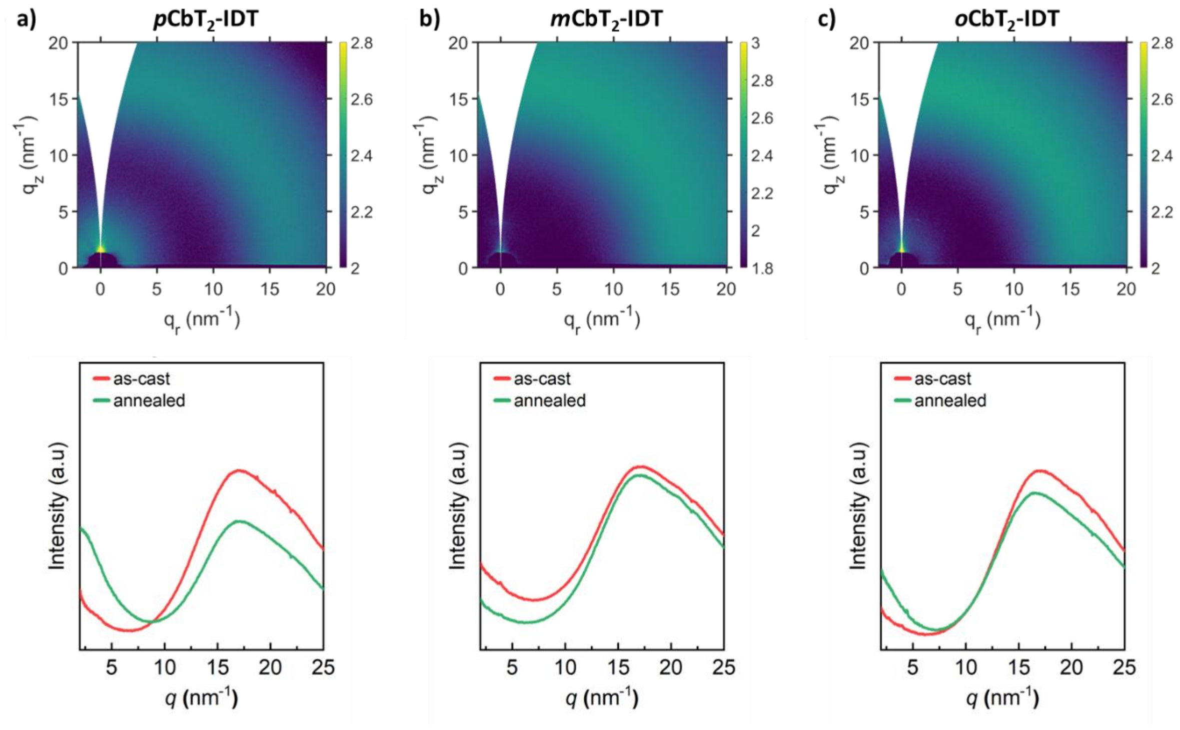

Figure S3. 2D GIWAXS (top) and cross-sectional (combined directional planes, bottom) diffractograms of a) *p*CbT$_2$-IDT, b) *m*CbT$_2$-IDT, and c) *o*CbT$_2$-IDT.

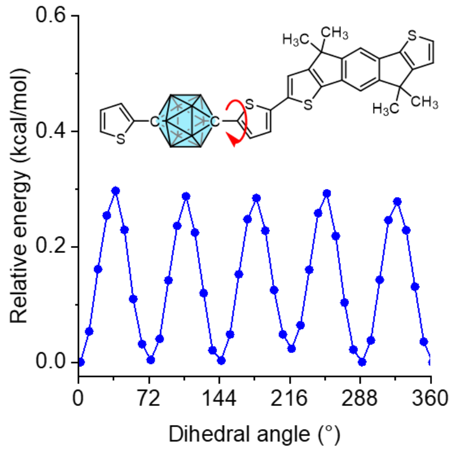

Figure S4. DFT calculated energetic barrier of rotation around the carborane-thiophene bond of a single *p*CbT$_2$-IDT repeat unit.

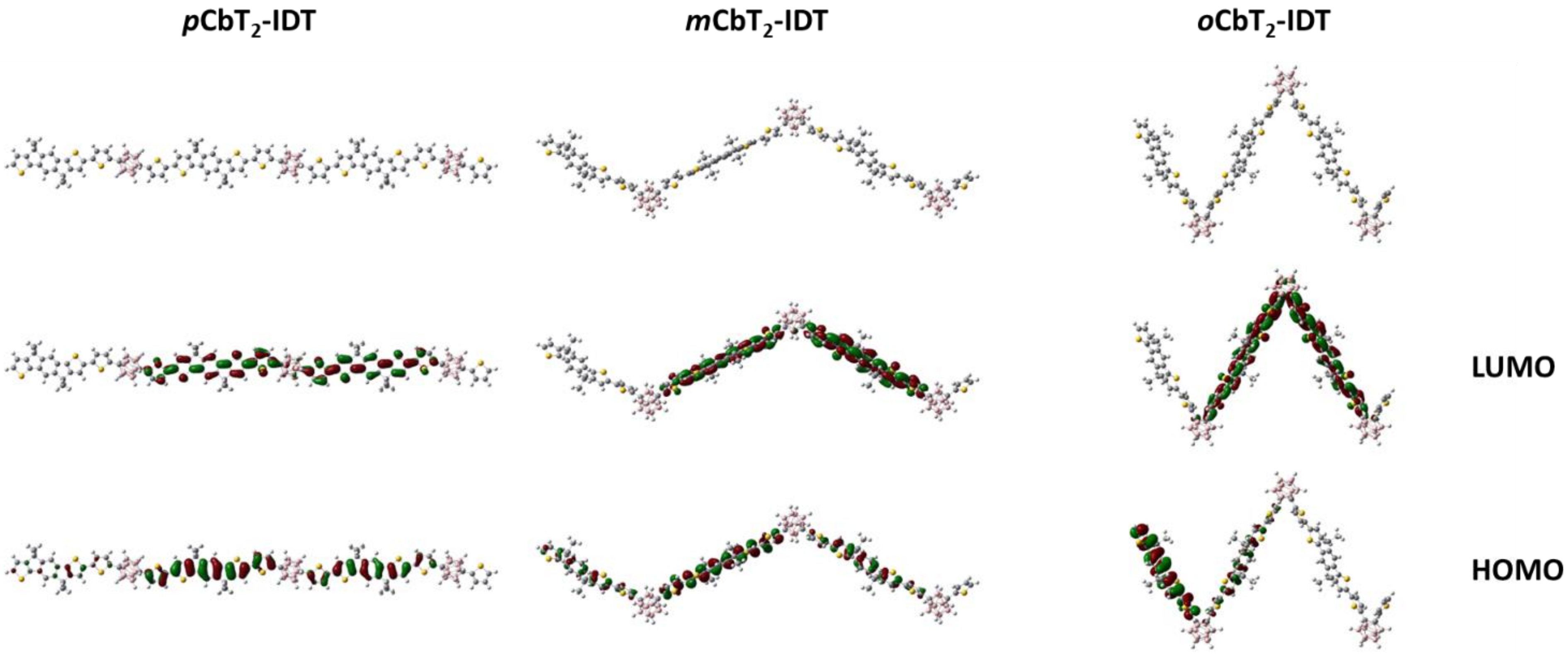

Figure S5. DFT modelling of the molecular structures and frontier molecular orbitals (FMOs) of the *p*CbT$_2$-IDT, *m*CbT$_2$-IDT, and *o*CbT$_2$-IDT polymers.

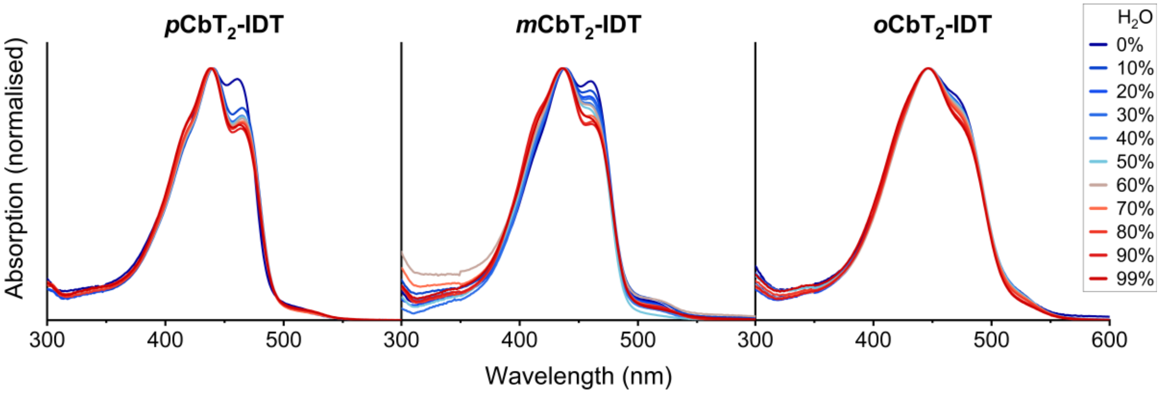

Figure S6. Absorption spectra of THF:water solutions (≤0.005 mg/mL) of respective polymer.

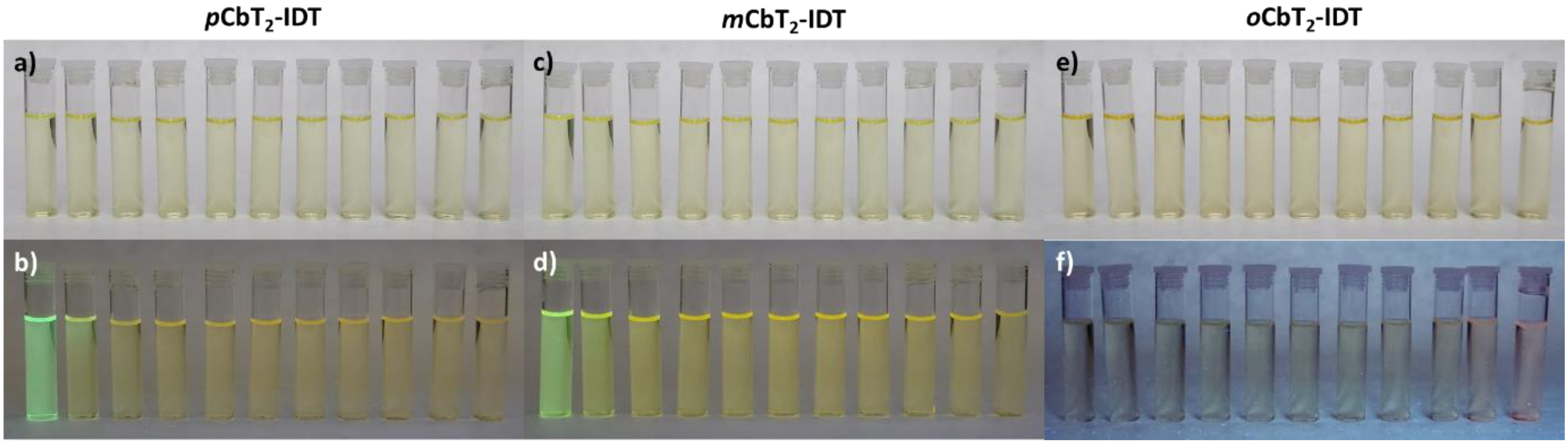

Figure S7. Photographs of vials containing THF:water solutions (0.005 mg/mL, 0, 10, ..., 90, 99 vol% water) of polymers *p*CbT2-IDT (a,b), *m*CbT2-IDT (c,d), and *o*CbT2-IDT (e,f) under ambient (a, c, e) and UV (λ_{ex} = 365 nm) (b, d, f) light.

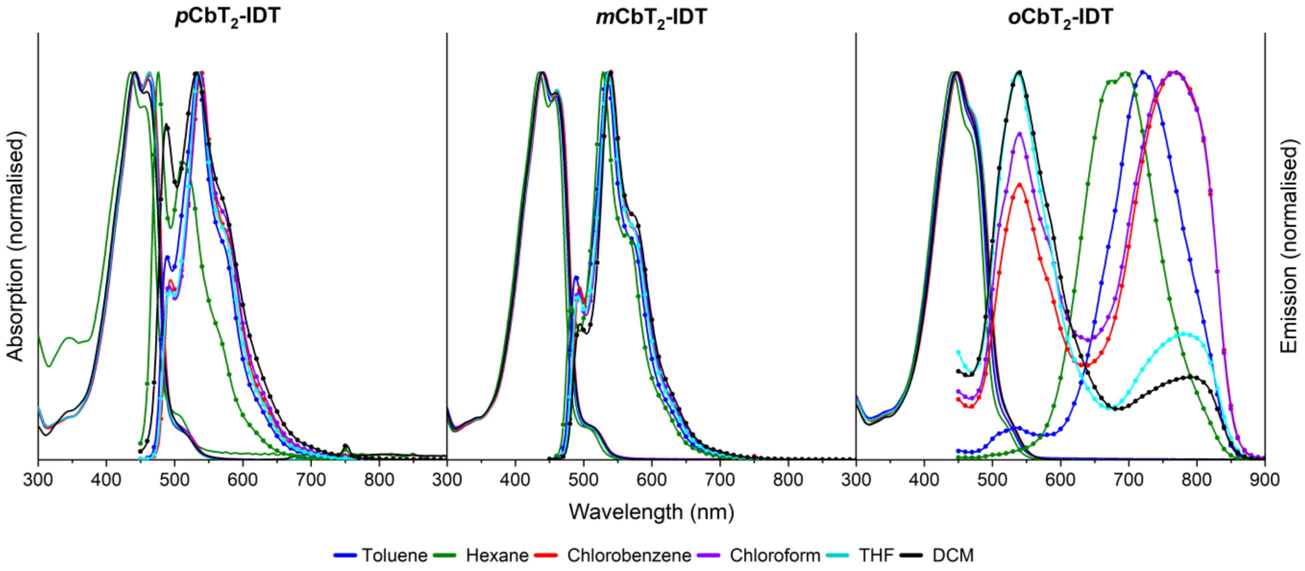

Figure S8. Absorption (solid lines) and PL (marked lines) spectra of polymer solutions (0.05 mg/mL or saturation) with various solvents.

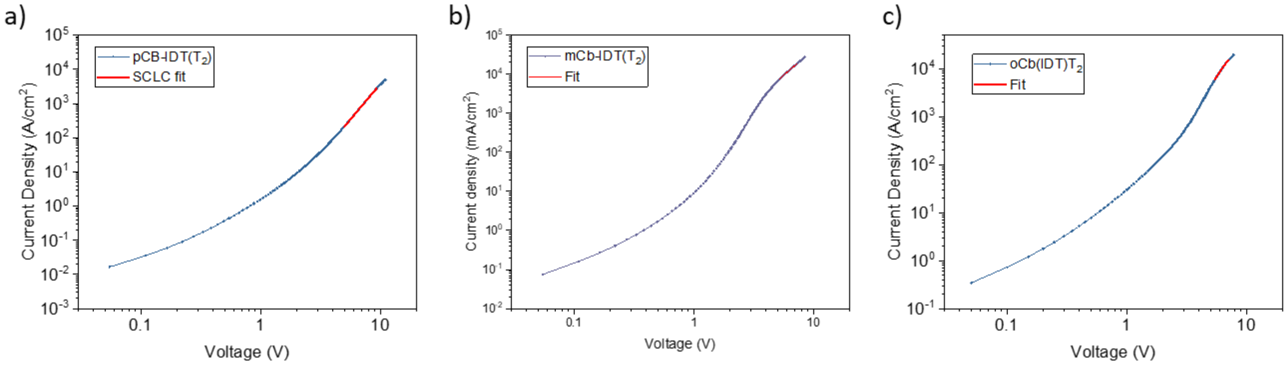

Figure S9. Output plots for SCLC devices of a) *p*CbT2-IDT, b) *m*CbT2-IDT, and c) *o*CbT2-IDT.

Table S1. Parameters used to determine solvent polarity.

	Toluene	Hexane	Chlorobenzene	Chloroform	THF	DCM
ε	2.38	2.02	5.62	4.81	7.58	8.93
n	1.4969	1.3749	1.5248	1.4458	1.4072	1.4241
Δf	0.013235	0.016143	0.142936	0.148295	0.209572	0.217137

Table S2. Excited state lifetimes as derived from TCSPC measurements (λ_{ex} = 375 nm).

	τ_1 (ns)	c_1 (%)	τ_2 (ns)	c_2 (%)	<τ> (ns)
*p*CbT$_2$-IDT[a]	0.86	99.9	11	0.1	0.87
*m*CbT$_2$-IDT[a]	0.91	99.9	13	0.1	0.92
*o*CbT$_2$-IDT (LE)[a]	0.39	64.7	1.0	35.3	0.61
*o*CbT$_2$-IDT (ICT)[b]	0.71	73.2	1.9	26.8	1.0

[a]Measured from THF solution. [b]Measured from 1:9 THF:water.

Synthesis

General

Chemicals were obtained from commercial sources and used without further purification, unless otherwise specified. Microwave reactions were run in a Biotage Initiator+ microwave reactor. Polymers were purified by preparative GPC on a Shimadzu UFLC prior to device fabrication.

1,12-di(5-(trimethylstannyl)thiophen-2-yl)-*para*/*meta*/*ortho*-carborane (*p*/*m*/*o*1)

Carboranyl monomers were synthesized and characterized as previously reported.[11]

Poly{[4,4,9,9-tetrahexadodecyl-4,9-dihydro-*s*-indaceno[1,2-*b*:5,6-*b*']dithiophen-2,7-diyl]-*alt*-5,5′-[1,12-bis(5-thiophen-2-yl)-*para*-carborane]} (*p*CbT$_2$-IDT)

1,12-Bis(5-(trimethylstannyl)thiophen-2-yl)-*para*-carborane (167.3 mg, 0.264 mmol, 1 eq.), 2,7-dibromide-4,4,9,9-tetrahexadodecyl-4,9-dihydro-*s*-indaceno[1,2-*b*:5,6-*b*']dithiophen (348.9 mg, 0.264 mmol, 1 eq.), tris(dibenzylideneacetone)dipalladium(0) (4.6 mg, 5.02×10^{-3} mmol, 0.02 eq.) and tri(o-tolyl)-phosphine (6.2 mg, 2.04×10^{-2} mmol, 0.08 eq.) were dissolved in dry degassed chlorobenzene (4.5 mL) under nitrogen in a microwave reaction tube. The mixture was heated in a microwave reactor with the following program: 120 °C for 2 minutes, 140 °C for 2 minutes, 160 °C for 2 minutes, 180 °C for 20 minutes. After cooling to room temperature, the crude product was precipitated into cold stirring methanol with 1 vol% HCl (2 M). The precipitate was filtered, and washed using Soxhlet extraction using methanol, acetone, and hexane sequentially. The hexane fraction was concentrated and precipitated into cold, well stirred methanol, and filtered to obtain 281 mg red-brown polymer (73 % yield):

^{1}H NMR (400 MHz, Chloroform-*d*) δ 7.19 (s, 2H), 6.97 (s, 2H), 6.85 (s, 2H), 6.69 (s, 2H), 3.63 – 2.01 (m, 10H), 2.01 – 0.99 (m, 120H), 0.88 (t, J = 6.6 Hz, 12H).

^{11}B NMR (128 MHz, Chloroform-*d*) δ -9.42 – -14.05 (br d, J = 168.6 Hz).

FTIR (neat) cm^{-1}: 2920 (s), 2850 (s), 2609 (s), 1604 (s), 1456 (m).

Poly{[4,4,9,9-tetrahexadodecyl-4,9-dihydro-*s*-indaceno[1,2-b:5,6-b']dithiophen-2,7-diyl]-*alt*-5,5′-[1,7-bis(5-thiophen-2-yl)-*meta*-carborane]} (*m*CbT$_2$-IDT)

The synthesis was identical to that of *p*CbT$_2$-IDT, to afford the product as an orange polymer (63 % yield):

^{1}H NMR (400 MHz, Chloroform-*d*) δ 7.21 (s, 2H), 7.02 (s, 2H), 6.94 (s, 4H), 3.96 – 2.14 (m, 10H), 1.90 (dt, J = 41.7, 10.0 Hz, 8H), 1.36 – 0.97 (m, 112H), 0.87 (t, J = 6.6 Hz, 12H).

^{11}B NMR (128 MHz, Chloroform-*d*) δ 5.08 – -25.33 (br s).

FTIR (neat) cm^{-1}: 2920 (s), 2850 (s), 2601 (m), 1604 (s), 1460 (m).

Poly{[4,4,9,9-tetrahexadodecyl-4,9-dihydro-*s*-indaceno[1,2-b:5,6-b']dithiophen-2,7-diyl]-*alt*-5,5′-[1,2-bis(5-thiophen-2-yl)-*ortho*-carborane]} (*o*CbT$_2$-IDT):

The synthesis was identical to that of *p*CbT$_2$-IDT, to afford the product as a red-brown polymer (67 % yield):

^{1}H NMR (400 MHz, Chloroform-*d*) δ 7.17 (s, 2H), 7.08 (d, J = 4.0 Hz, 2H), 7.02 (s, 2H), 6.89 (d, J = 4.0 Hz, 2H), 2.81 (m, 10H), 1.86 (dt, J = 48.5, 12.7 Hz, 8H), 1.34 – 0.98 (m, 112H), 0.87 (t, J = 6.8 Hz, 12H).

^{11}B NMR (128 MHz, Chloroform-*d*) δ 19.39 – -7.31 (br s), -7.70 – -40.15 (br s).

FTIR (neat) cm^{-1}: 2920 (s), 2850 (s), 2592 (m), 2576 (m), 1604 (s), 1462 (m).

Characterization

Poly{[4,4,9,9-tetrahexadodecyl-4,9-dihydro-*s*-indaceno[1,2-*b*:5,6-*b'*]dithiophen-2,7-diyl]-*alt*-5,5′-[1,12-bis(5-thiophen-2-yl)-*para*-carborane]} (*p*CbT_2-IDT)

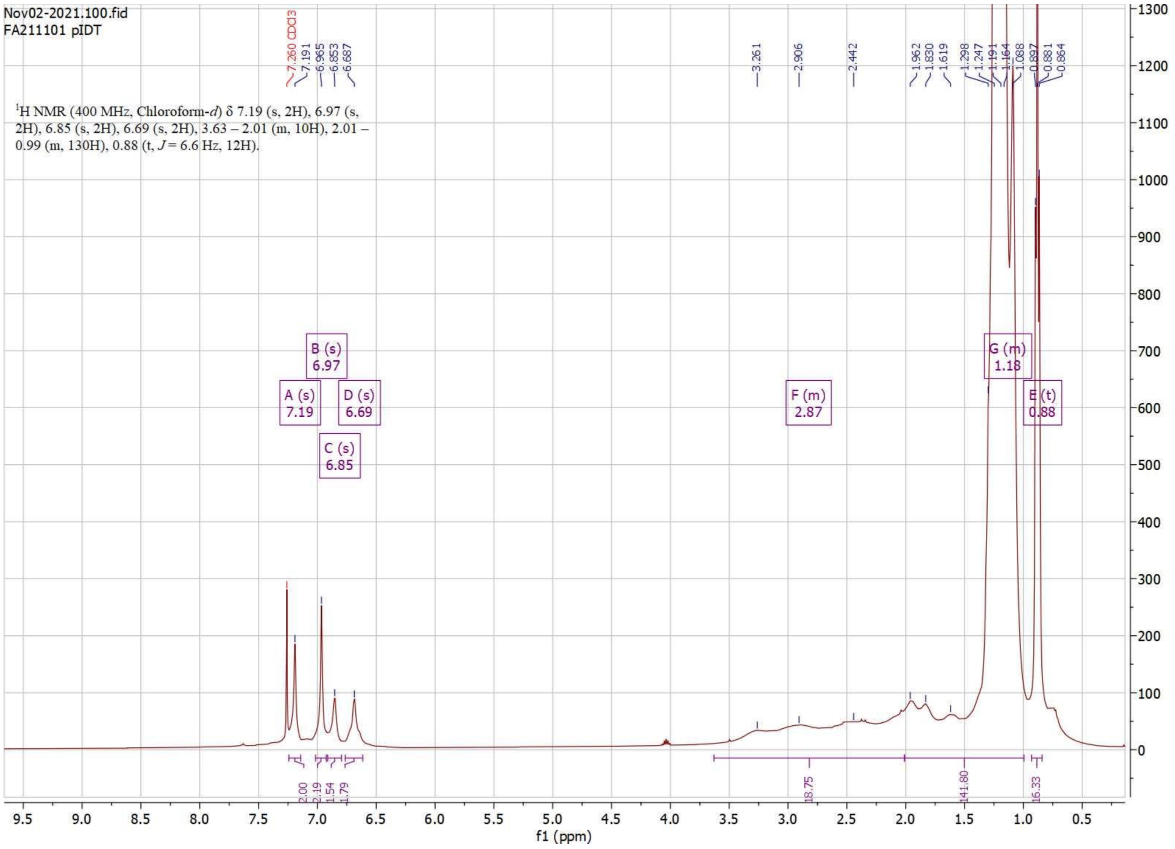

Figure S10. ^{1}H NMR spectrum of *p*CbT_2-IDT.

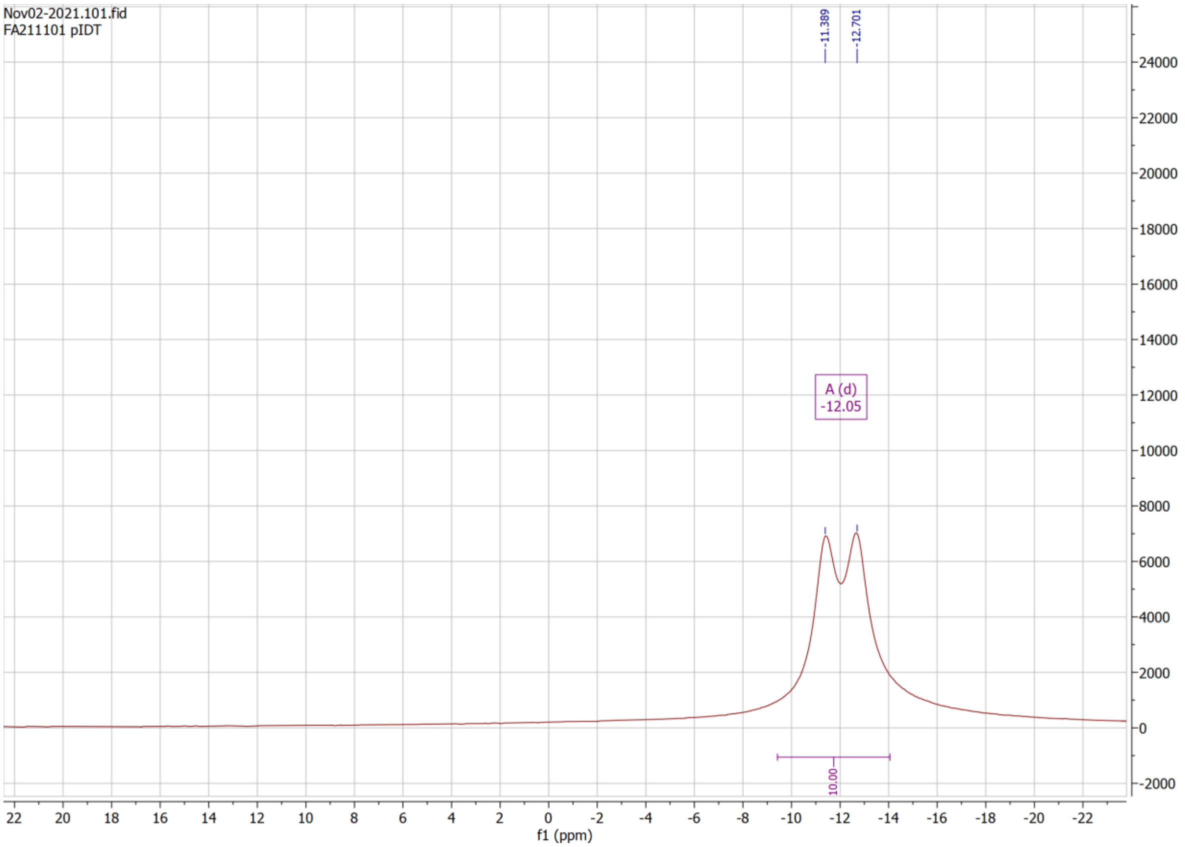

Figure S11. ^{11}B NMR spectrum of *p*CbT$_2$-IDT.

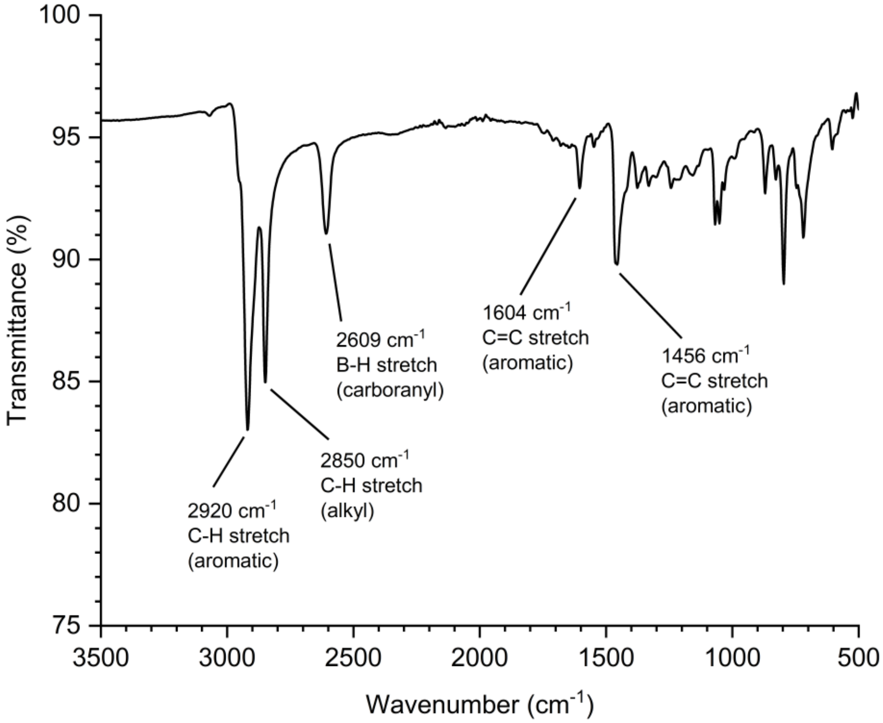

Figure S12. FTIR spectrum of *p*CbT$_2$-IDT.

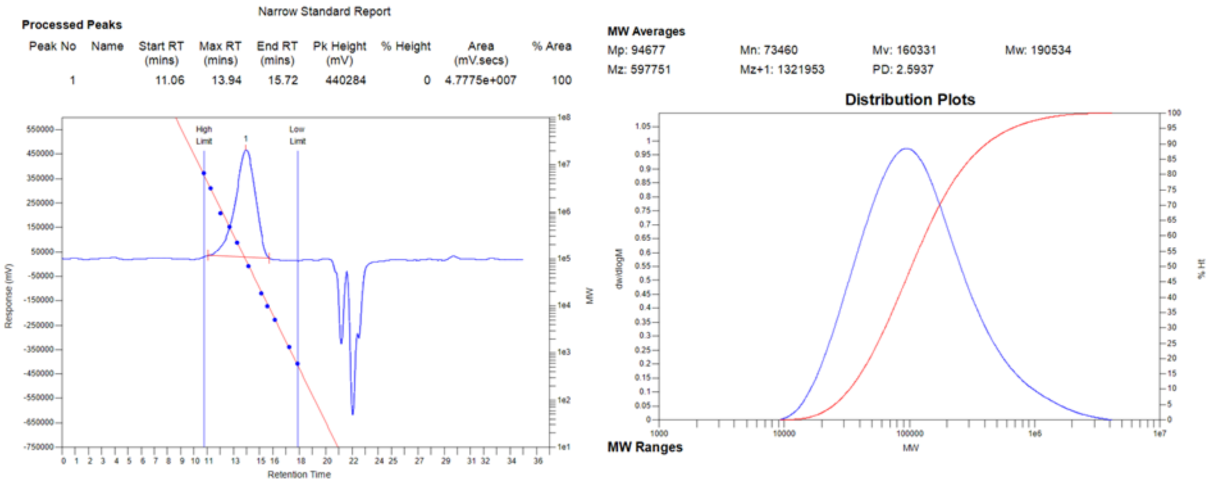

Figure S13. Analytical GPC trace (left) and peak report (right) of *p*CbT$_2$-IDT.

Poly{[4,4,9,9-tetrahexadodecyl-4,9-dihydro-*s*-indaceno[1,2-*b*:5,6-*b*′]dithiophen-2,7-diyl]-*alt*-5,5′-[1,7-bis(5-thiophen-2-yl)-*meta*-carborane]} (*m*CbT$_2$-IDT)

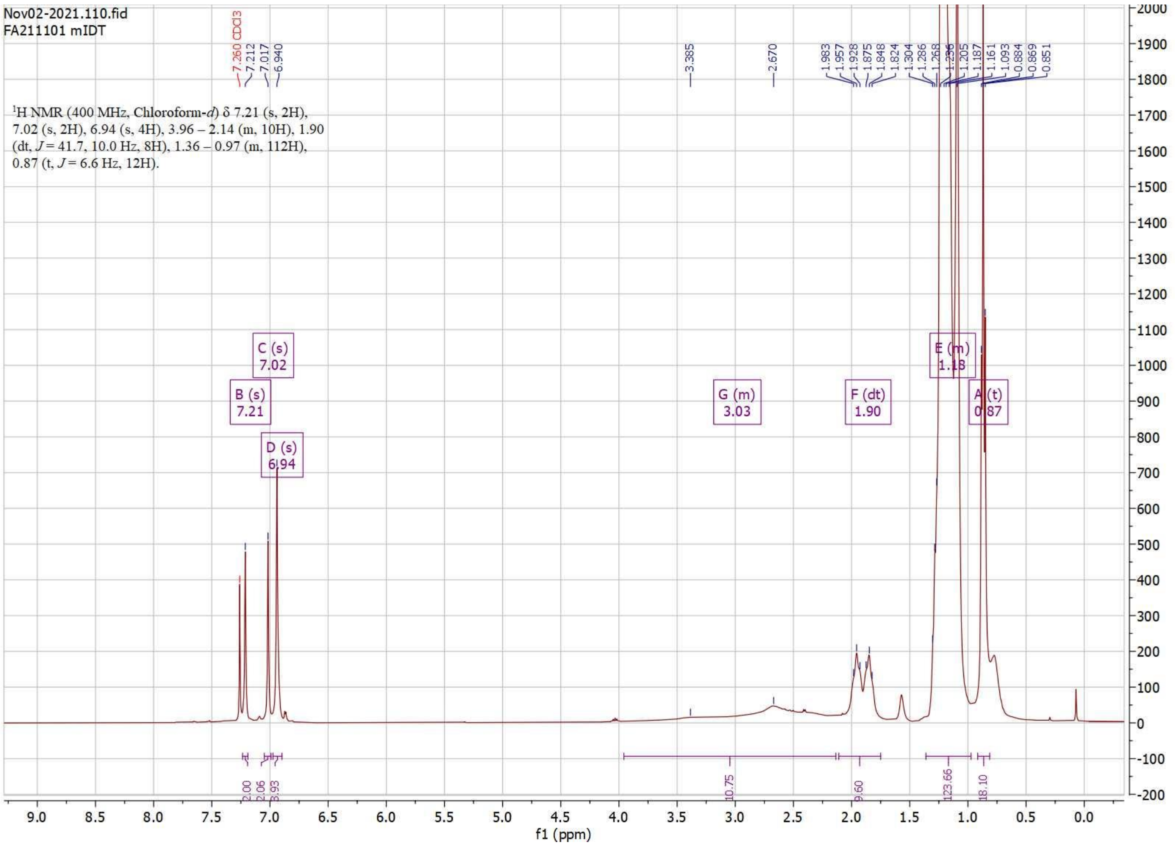

Figure S14. ^{1}H NMR spectrum of *m*CbT$_2$-IDT.

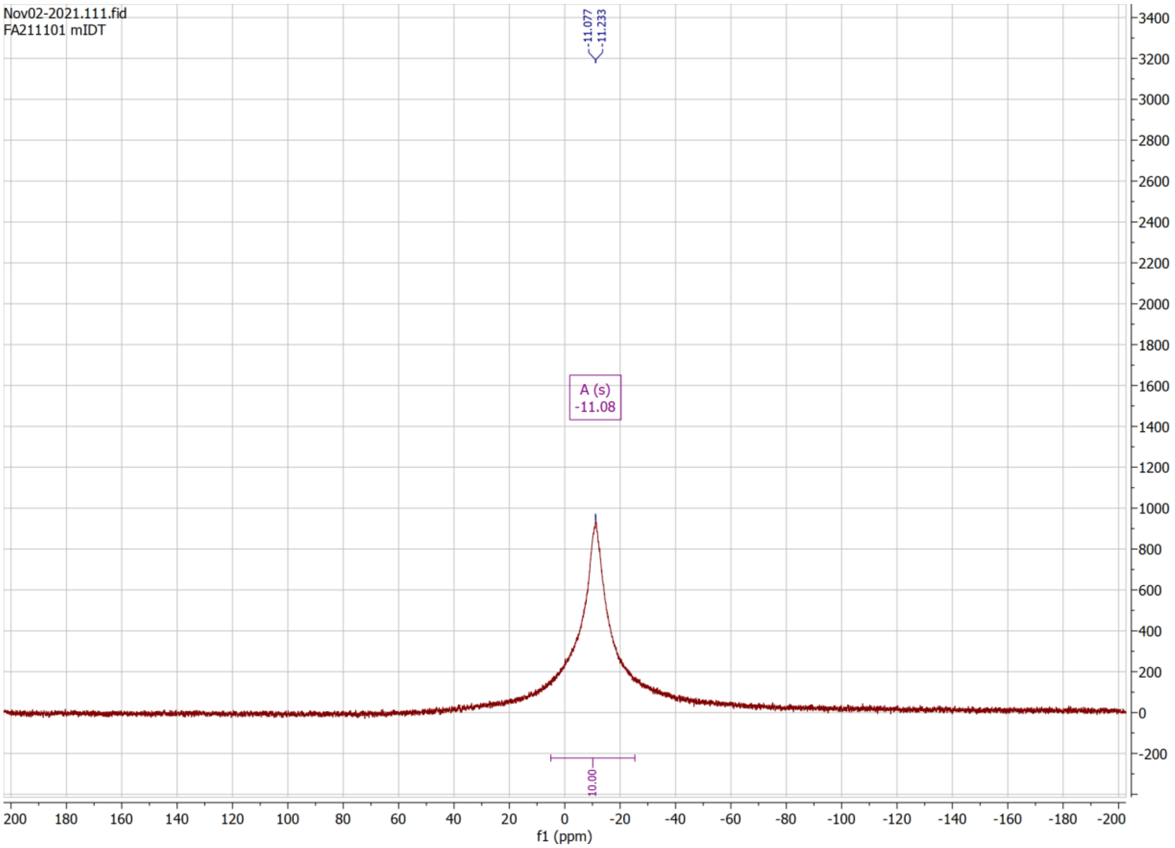

Figure S15. ^{11}B NMR spectrum of *m*CbT$_2$-IDT.

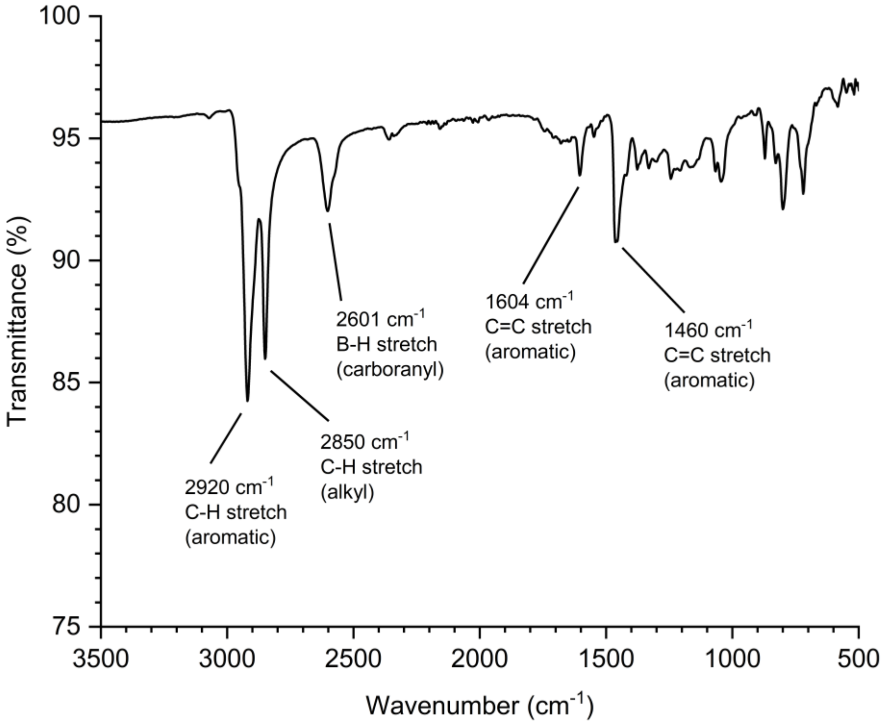

Figure S16. FTIR spectrum of *m*CbT$_2$-IDT.

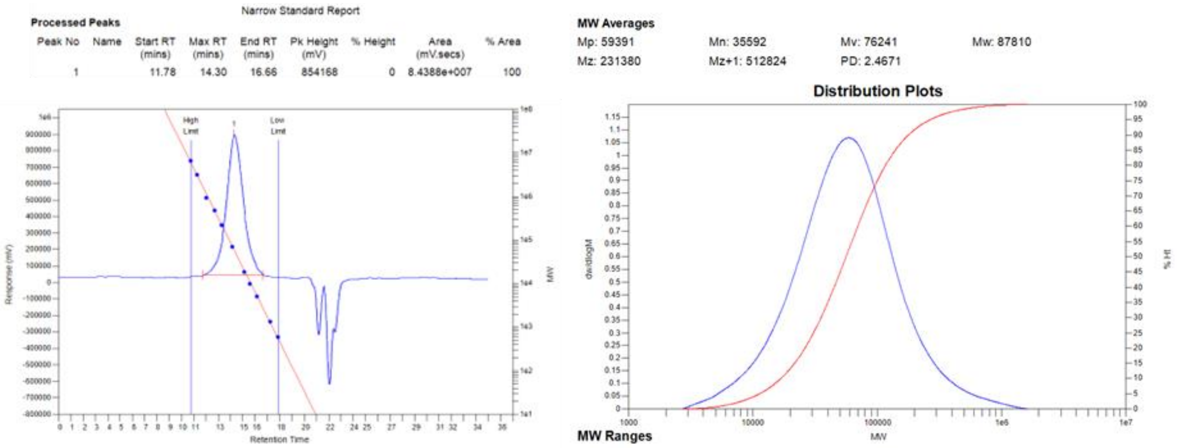

Figure S17. Analytical GPC trace (left) and peak report (right) of *m*CbT$_2$-IDT.

Poly{[4,4,9,9-tetrahexadodecyl-4,9-dihydro-*s*-indaceno[1,2-*b*:5,6-*b*']dithiophen-2,7-diyl]-*alt*-5,5′-[1,2-bis(5-thiophen-2-yl)-*ortho*-carborane]} (*o*CbT$_2$-IDT)

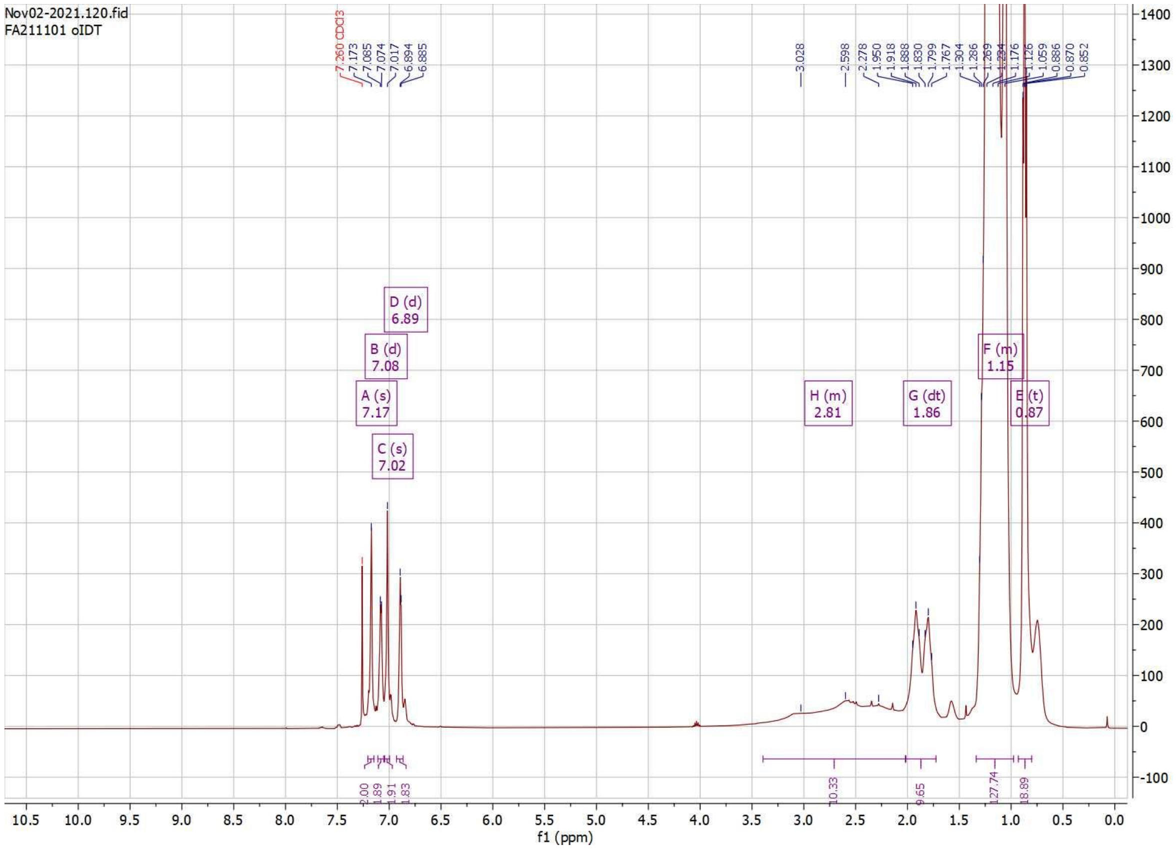

Figure S18. ^{1}H NMR spectrum of *o*CbT$_2$-IDT.

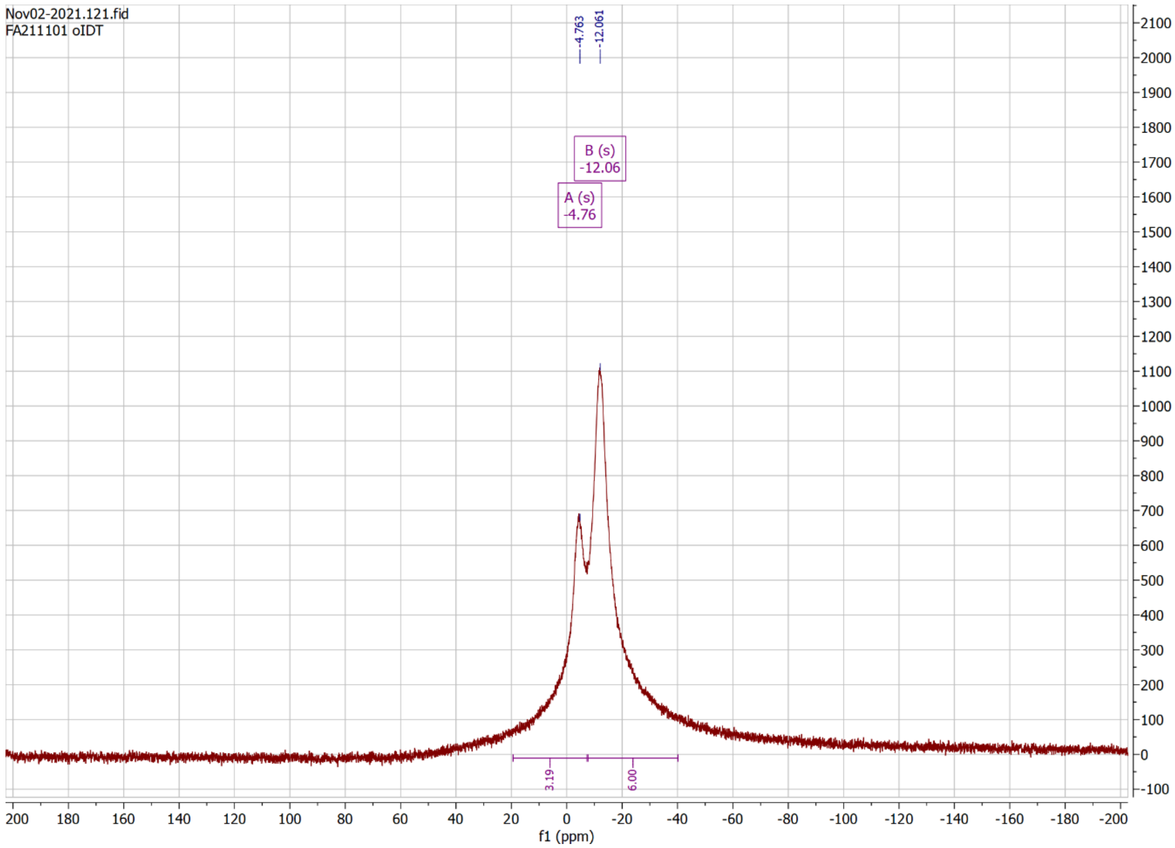

Figure S19. ^{11}B NMR spectrum of *o*CbT$_2$-IDT.

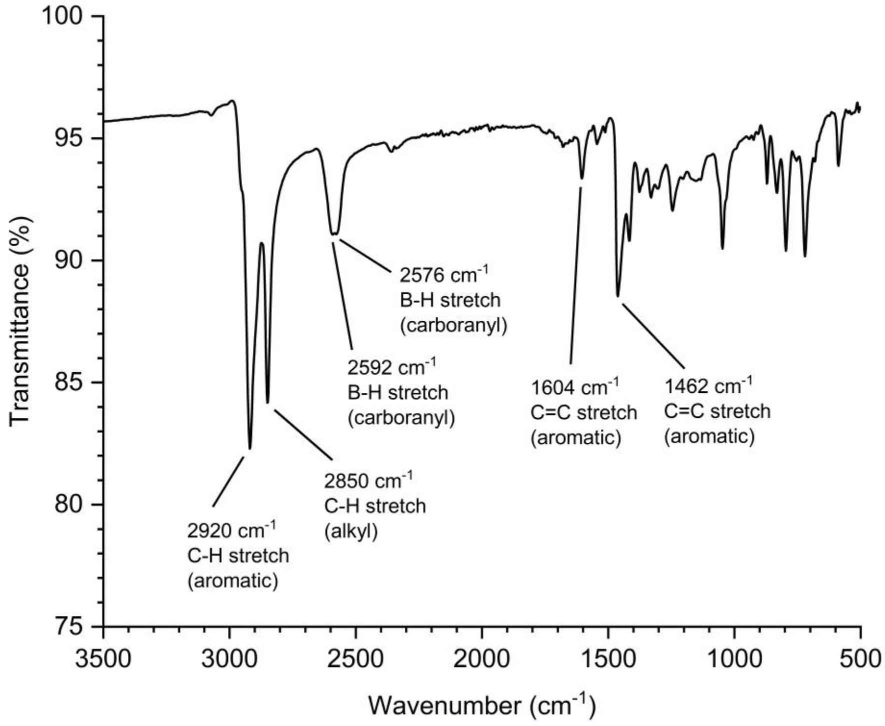

Figure S20. FTIR spectrum of *o*CbT$_2$-IDT.

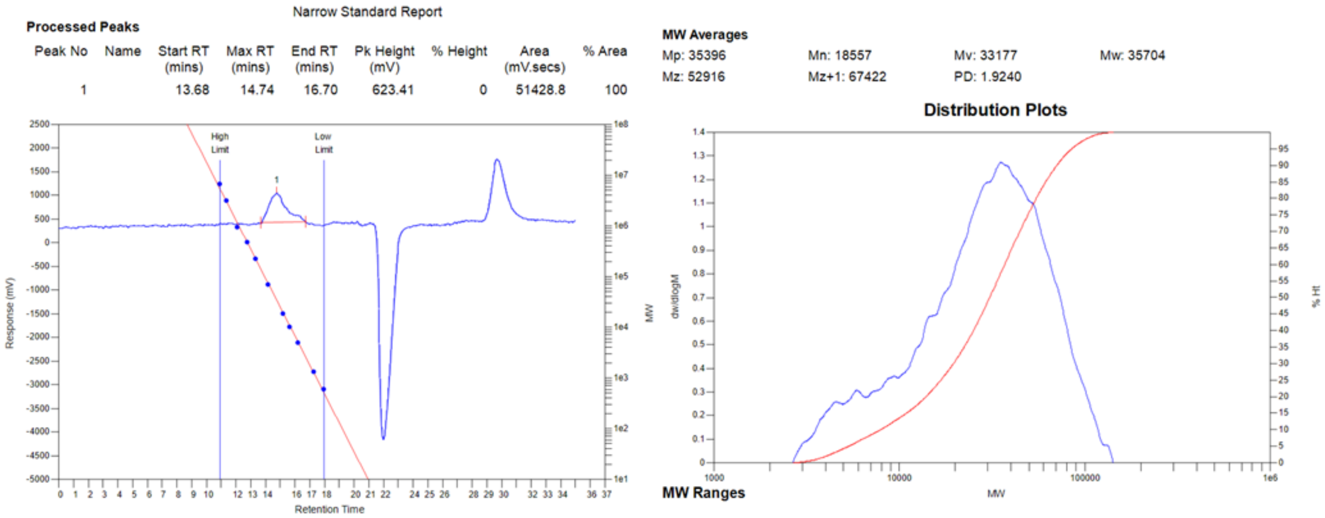

Figure S21. Analytical GPC trace (left) and peak report (right) of *o*CbT$_2$-IDT.

References

(1) Frisch, M. J.; Trucks, G. W.; Schlegel, H. B.; Scuseria, G. E.; Robb, M. A.; Cheeseman, J. R.; Scalmani, G.; Barone, V.; Petersson, G. A.; Nakatsuji, H.; Li, X.; Caricato, M.; Marenich, A. V.; Bloino, J.; Janesko, B. G.; Gomperts, R.; Mennucci, B.; Hratchian, H. P.; Ortiz, J. V.; Izmaylov, A. F.; Sonnenberg, J. L.; Williams-Young, D.; Ding, F.; Lipparini, F.; Egidi, F.; Goings, J.; Peng, B.; Petrone, A.; Henderson, T.; Ranasinghe, D.; Zakrzewski, V. G.; Gao, J.; Rega, N.; Zheng, G.; Liang, W.; Hada, M.; Ehara, M.; Toyota, K.; Fukuda, R.; Hasegawa, J.; Ishida, M.; Nakajima, T.; Honda, Y.; Kitao, O.; Nakai, H.; Vreven, T.; Throssell, K.; Montgomery, J. A. . J.; Peralta, J. E.; Ogliaro, F.; Bearpark, M. J.; Heyd, J. J.; Brothers, E. N.; Kudin, K. N.; Staroverov, V. N.; Keith, T. A.; Kobayashi, R.; Normand, J.; Raghavachari, K.; Rendell, A. P.; Burant, J. C.; Iyengar, S. S.; Tomasi, J.; Cossi, M.; Millam, J. M.; Klene, M.; Adamo, C.; Cammi, R.; Ochterski, J. W.; Martin, R. L.; Morokuma, K.; Farkas, O.; Foresman, J. B.; Fox, D. J. Gaussian 16, Revision C.01. Gaussian, Inc.: Wallingford CT 2016.

(2) Becke, A. D. Density-functional Thermochemistry. III. The Role of Exact Exchange. *J. Chem. Phys.* **1993**, *98* (7), 5648–5652. https://doi.org/10.1063/1.464913.

(3) Seixas de Melo, J. S.; Pina, J.; Dias, F. B.; Maçanita, A. L. Experimental Techniques for Excited State Characterisation. In *Applied Photochemistry*; Springer Netherlands: Dordrecht, 2013; pp 533–585. https://doi.org/10.1007/978-90-481-3830-2_15.

(4) Reynolds, G. A.; Drexhage, K. H. New Coumarin Dyes with Rigidized Structure for Flashlamp-Pumped Dye Lasers. *Opt. Commun.* **1975**, *13* (3), 222–225. https://doi.org/10.1016/0030-4018(75)90085-1.

(5) Sens, R.; Drexhage, K. H. Fluorescence Quantum Yield of Oxazine and Carbazine Laser Dyes. *J. Lumin.* **1981**, *24–25*, 709–712. https://doi.org/10.1016/0022-2313(81)90075-2.

(6) Jones, G.; Jackson, W. R.; Choi, C. Y.; Bergmark, W. R. Solvent Effects on Emission Yield and Lifetime for Coumarin Laser Dyes. Requirements for a Rotatory Decay Mechanism. *J. Phys. Chem.* **1985**, *89* (2), 294–300. https://doi.org/10.1021/j100248a024.

(7) Critchfield, F. E.; Gibson, J. A.; Hall, J. L. Dielectric Constant and Refractive Index from 20 to 35° and Density at 25° for the System Tetrahydrofuran—Water 1. *J. Am. Chem. Soc.* **1953**, *75* (23), 6044–6045. https://doi.org/10.1021/ja01119a509.

(8) de Mello, J. C.; Wittmann, H. F.; Friend, R. H. An Improved Experimental Determination of External Photoluminescence Quantum Efficiency. *Adv. Mater.* **1997**, *9* (3), 230–232. https://doi.org/10.1002/adma.19970090308.

(9) Magde, D.; Brannon, J. H.; Cremers, T. L.; Olmsted, J. Absolute Luminescence Yield of Cresyl Violet. A Standard for the Red. *J. Phys. Chem.* **1979**, *83* (6), 696–699. https://doi.org/10.1021/j100469a012.

(10) Isak, S. J.; Eyring, E. M. Cresyl Violet Chemistry and Photophysics in Various Solvents and Micelles. *J. Photochem. Photobiol. A Chem.* **1992**, *64* (3), 343–358. https://doi.org/10.1016/1010-6030(92)85008-I.

(11) Aniés, F.; Qiao, Z.; Nugraha, M. I.; Basu, A.; Anthopoulos, T. D.; Gasparini, N.; Heeney, M. N-Type Polymer Semiconductors Incorporating Para, Meta, and Ortho-Carborane in the Conjugated Backbone. *Polymer (Guildf).* **2022**, *240*, 124481. https://doi.org/10.1016/j.polymer.2021.124481.
